# Supplementary material for: Positional programs in early murine facial development and their role in human facial shape variability
Source: Nat Commun. 2025 Nov 18;16:10112. doi: 10.1038/s41467-025-66017-y (PMC12627740; doi:10.1038/s41467-025-66017-y)
Supplement: Supplementary file 1 — Supplementary Information [file 41467_2025_66017_MOESM1_ESM.pdf]

Supplementary Figure 1. Dataset summary

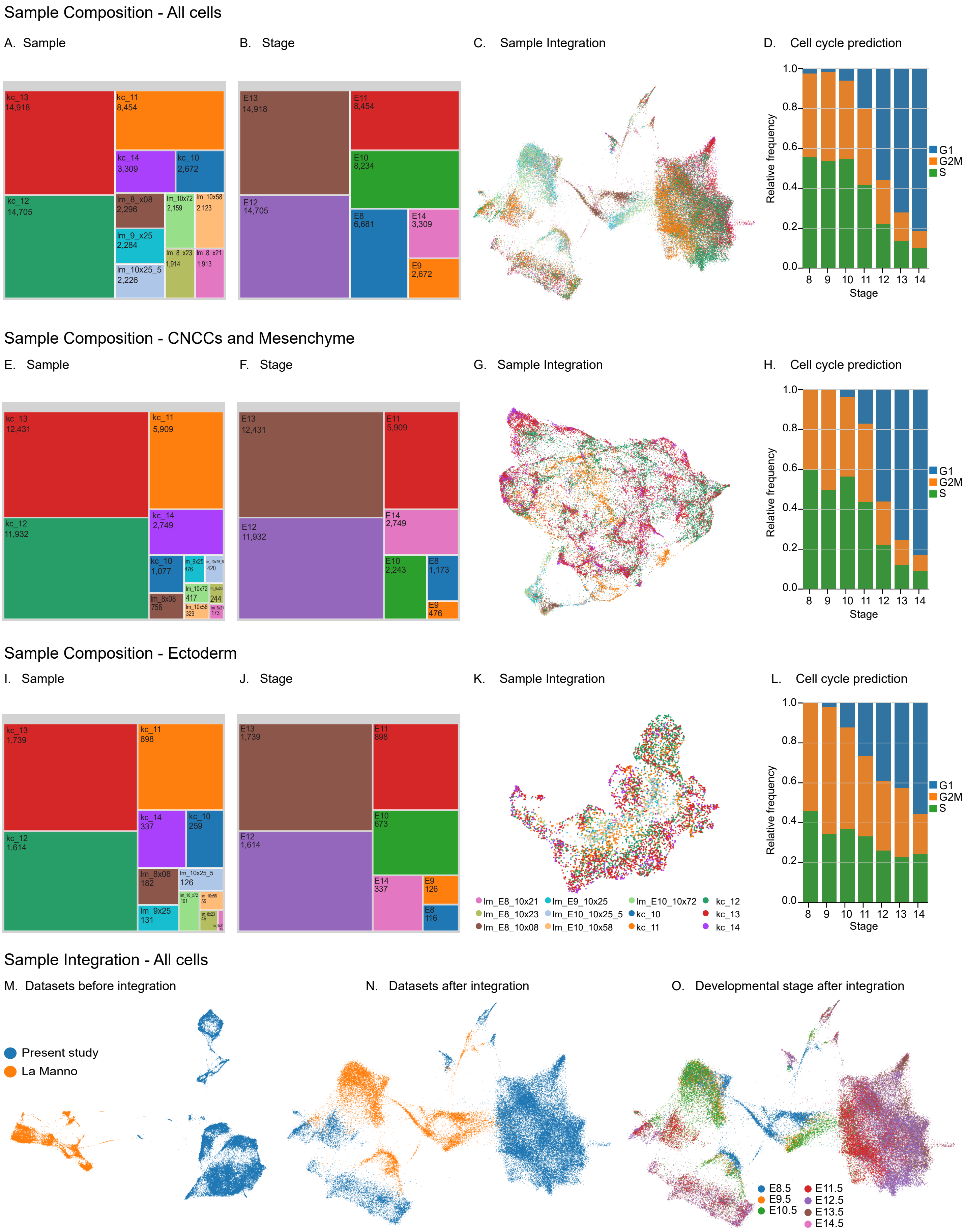

Supplementary Figure 1. **Single-cell data set overview**.

A) Contribution of cells per sample to the total dataset. B) Contribution of cells per stage to the total dataset. C) UMAP embedding of the total dataset colored by sample showing the integration of the different samples. D) Stacked bar plot of the predicted cell cycle phases per stage. Cell cycle predictions were calculated using scanpy.tl.score_genes_cell_cycle and aggregated per stage. E, F, G, H) Similar to the previous figures, but for the subset of neural crest and mesenchymal cells. I, J, K, L) Similar to the previous figures, but for the subset of the ectoderm population. Sample names and color legend in K refer to the data set (Present study or La Manno) and batch (12 batches) of origin for cells in UMAPs in C, G and K. To demonstrate proper sample integration, integrated UMAPs for all cells before (M) and after (N) integration are provided. Distribution of cells in the UMAP after integration (N) corresponds to the development stage of origin (O), where early stages (E8.5-E10.5) that are located at the center, belong to La Manno data set, and later stages (E10.5-E14.5) belonging to our original data set (Present study), are located towards the periphery of the UMAP.

Supplementary Figure 2

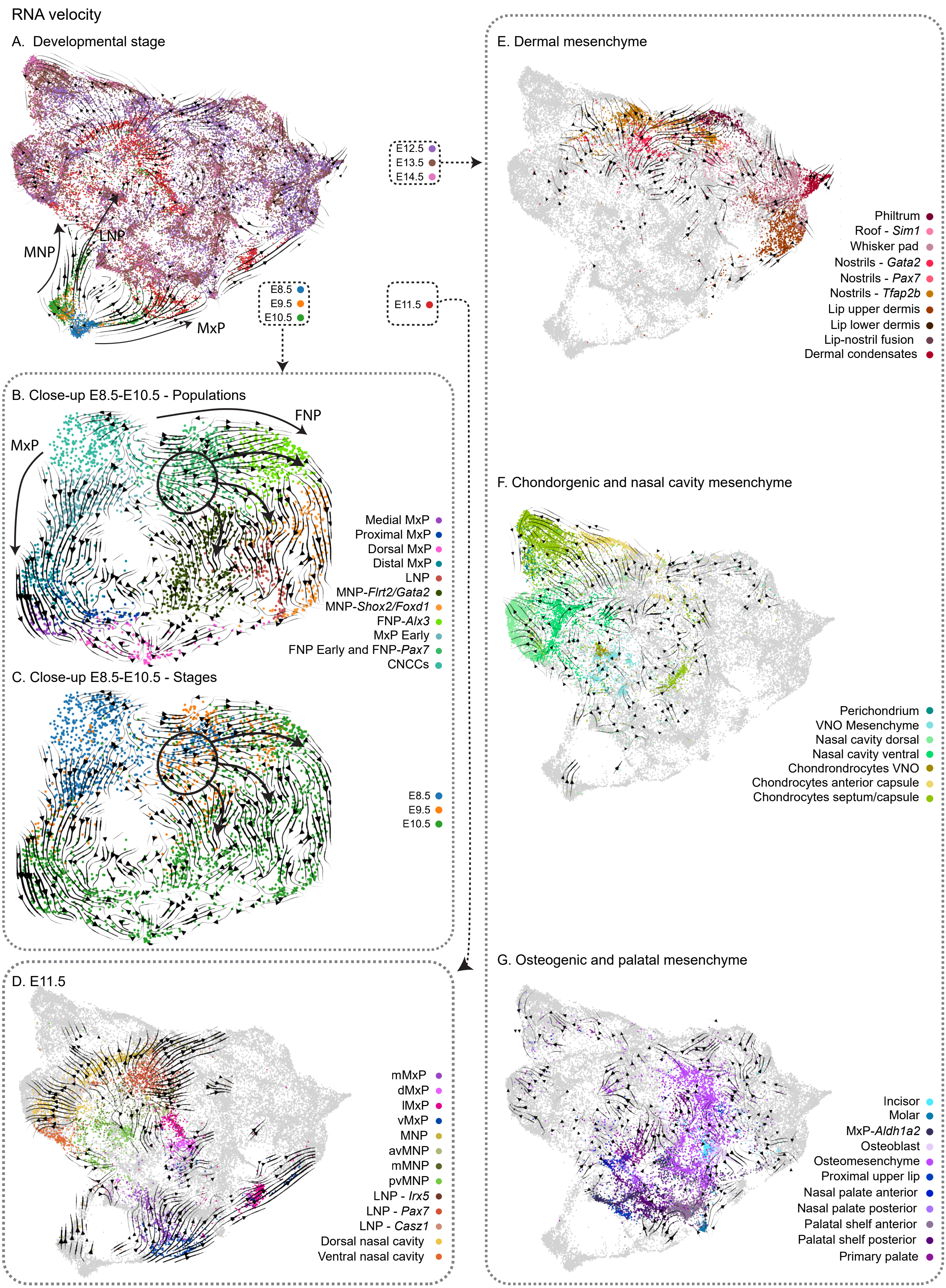

Supplementary Figure 2. RNA velocity analysis

A) RNA velocity vectors that predict developmental trajectories are overlaid on integrated UMAP and colored according to developmental stage. B-C) RNA velocity close-up for integrated stages E8.5-E10.5 on UMAP colored by cluster (B) and developmental stage (C). Black circles and arrows highlight the split of trajectories in the FNP as early as E9.5. D) RNA velocity for E11.5 on UMAP colored by cluster. E-G) RNA velocity for E) dermal mesenchyme populations, F) chondrogenic and nasal cavity mesenchyme populations, and G) osteogenic and palatal mesenchyme populations.

LNP, lateral nasal prominence; MNP, medial nasal prominence; MxP, maxillary prominence.

Supplementary Figure 3

Anatomical directional terms used to name the positional clusters

A. Frontal view, anterior/distal section

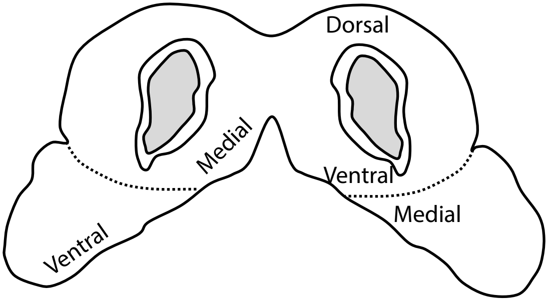

B. Frontal view, posterior/proximal section

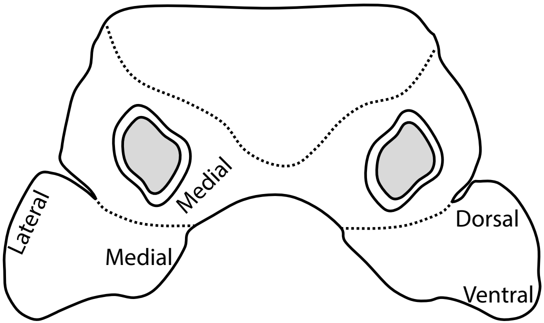

C. Lateral view

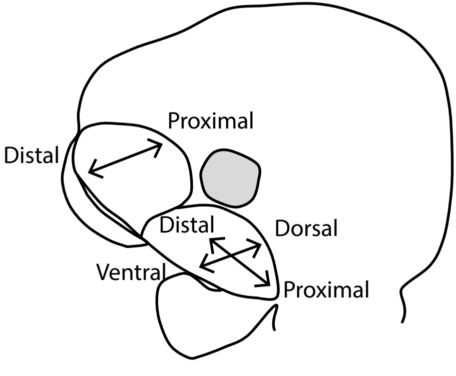

D. Frontal view - Palatal shelves

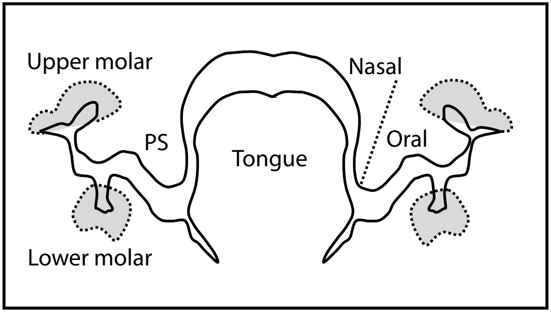

Supplementary Figure 3. **Anatomical directional terms used to name the positional clusters.**

A-D) Schematic representations of an E10.5-E11.5 mouse embryonic face, describing the positional terms used to name the mesenchymal clusters in frontal anterior (A) and posterior (B) sections, as well as in a lateral view (C). D) Frontal section of an E13.5 embryo oral cavity, at the molar level, shows the division into nasal and oral sides of the palatal shelves (PS).

Supplementary Figure 4

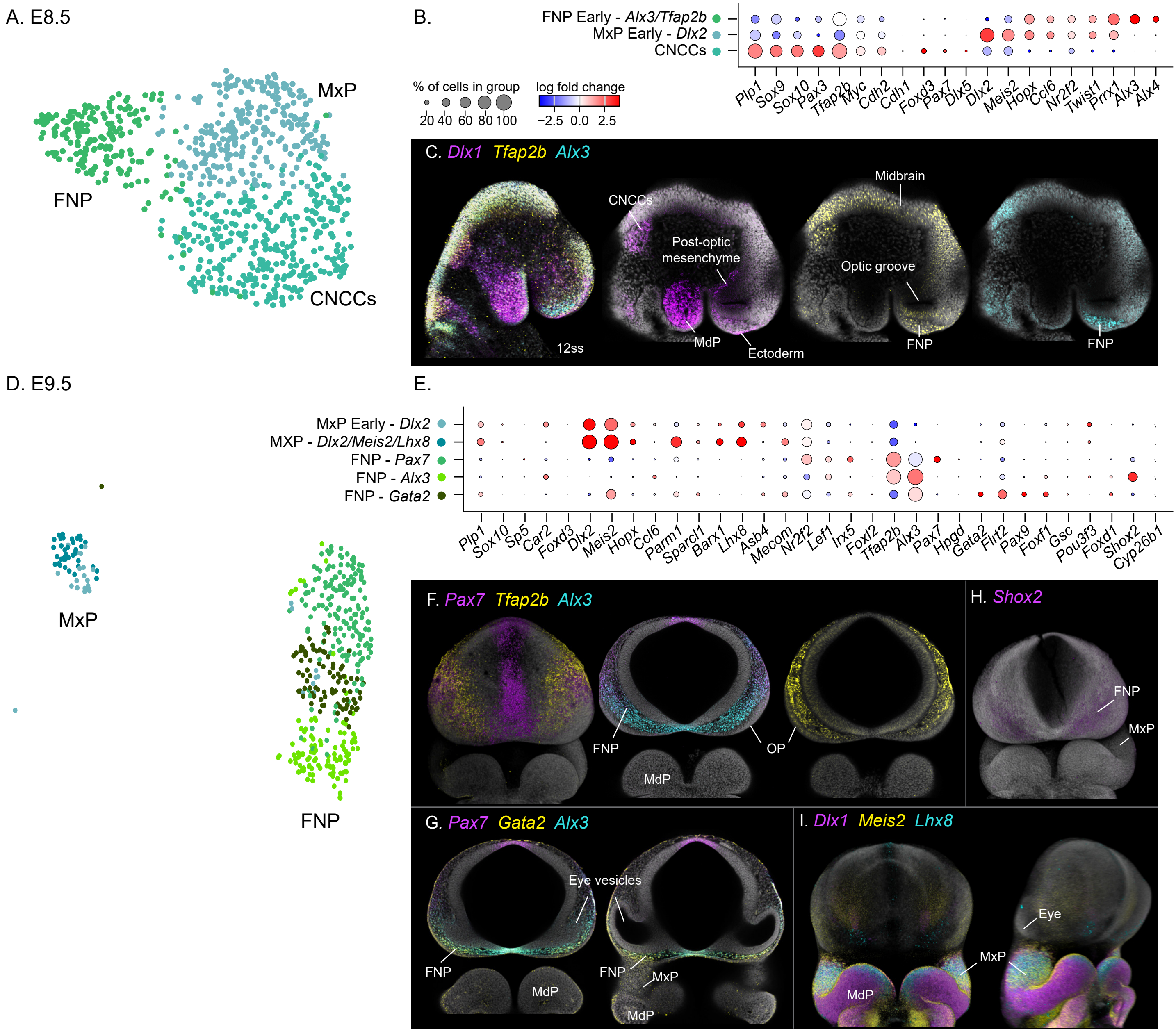

Supplementary Figure 4. Cluster annotation for E8.5 and E9.5

A) UMAP showing cellular composition at E8.5. B) Dot plot of selected genes used for cluster identification at E8.5. C) Multiplex *in situ* hybridization (HCR) showing the expression patterns of marker genes in whole mount and cross sections at E8.5. D) UMAP showing cellular composition at E9.5. E) Dot plot of selected genes used for cluster identification at E9.5, including early positional markers. F-I) Multiplex *in situ* hybridization (HCR) showing the expression pattern of early positional genes in whole mount and cross sections at E9.5.

CNCCs, cranial neural crest cells; FNP, frontonasal process; MdP, mandibular prominence; MxP, maxillary prominence; OP, olfactory placode.

Supplementary Figure 5

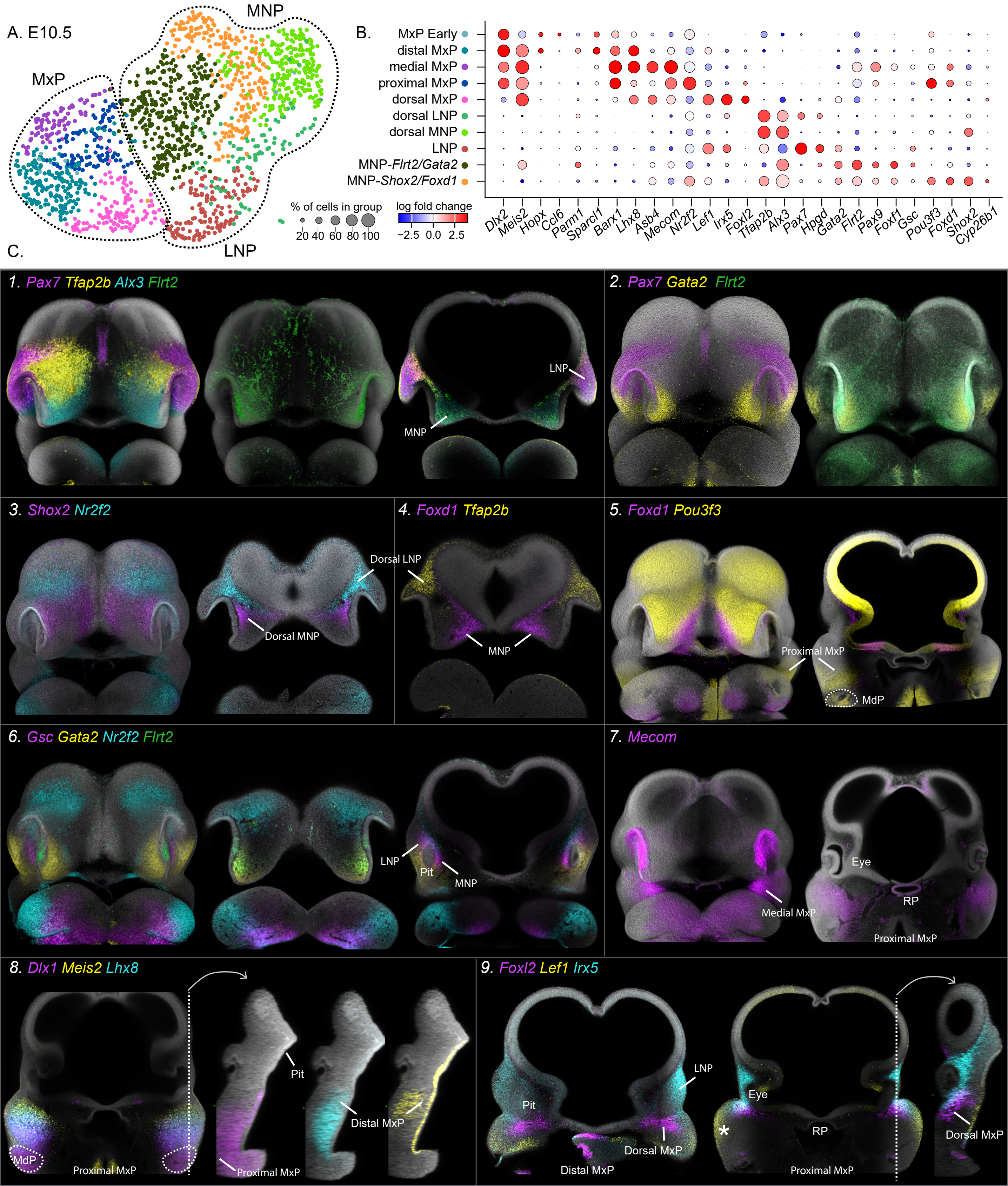

Supplementary Figure 5. Cluster annotation for E10.5

A) UMAP showing cellular composition at E10.5. Dotted lines delineate frontonasal prominence-derived (LNP and MNP) and maxillary prominence-derived (MxP) clusters. B) Dot plot for selected genes used for cluster identification, including early positional markers. C) Multiplex *in situ* hybridization (HCR) showing the expression patterns of early positional genes in whole mount and cross sections. Broken lines in C8-9 mark the level of the section shown in the corresponding panel. The asterisk in C9 indicates the incipient expression of *Lef1* at the lateral-posterior area of the MxP.

LNP, lateral nasal prominence; MdP, mandibular prominence; MNP, medial nasal prominence; MxP, maxillary prominence; Pit, nasal pit; RP, Rathke's pouch.

Supplementary Figure 6

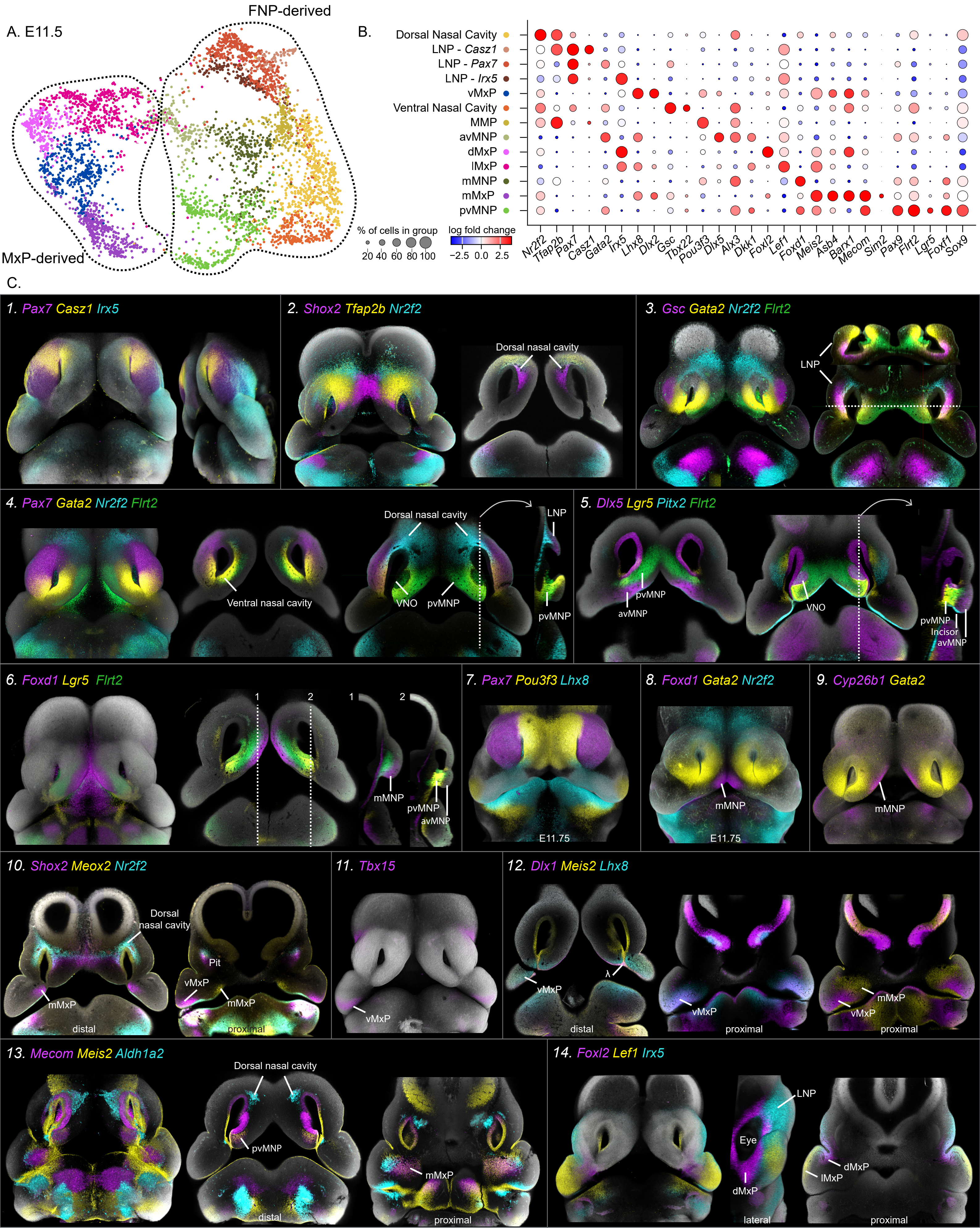

Supplementary Figure 6. Cluster annotation for E11.5

A) UMAP showing cellular composition at E11.5. B) Dot plot for selected genes used for cluster identification, including early positional markers. C) Multiplex *in situ* hybridization (HCR) showing the expression pattern of early positional genes in whole mount and cross sections. Broken lines in C3-C6 mark the level of the section shown in the corresponding panel. Panels C7 and C8 show E11.75 embryos.

LNP, lateral nasal prominence; MNP, medial nasal prominence; MNP, medial nasal prominence; avMNP, anteroventral medial nasal prominence; pvMNP, posteroventral medial nasal prominence; mMNP, medial medial nasal prominence; mMxP, medial maxillary prominence, dMxP, dorsal maxillary prominence; lMxP, lateral maxillary prominence; vMxP, ventral maxillary prominence; Pit, nasal pit; RP, Rathke's pouch; VNO, vomeronasal organ.

Supplementary Figure 7

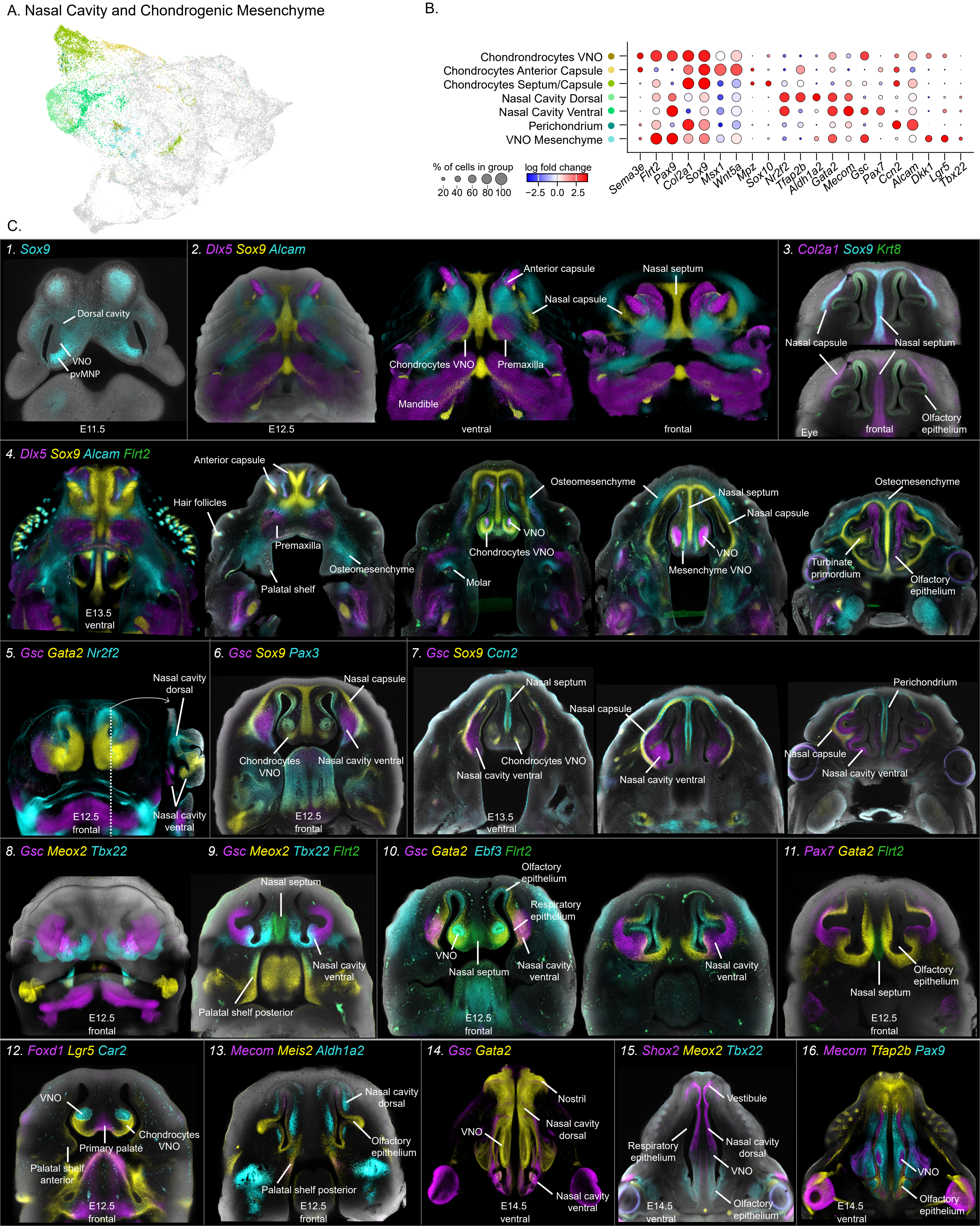

Supplementary Figure 7. Cluster identity and validation for chondrogenic and nasal cavity mesenchyme from E12.5 to E14.5.

A) Integrated UMAP highlighting the cells representing chondrocytes and the mesenchyme around the nasal cavities, marked by the expression of *Sox9*. B) Dot plot of selected genes used for cluster identification, including early positional and canonical cell type markers. C) Multiplex *in situ* hybridization (HCR) showing the expression patterns of selected marker genes and the location of clusters listed in (B) in whole mount and cross sections. The vertical broken line in C5 marks the level of the section shown in the corresponding panel.

VNO, vomeronasal organ.

Supplementary Figure 8

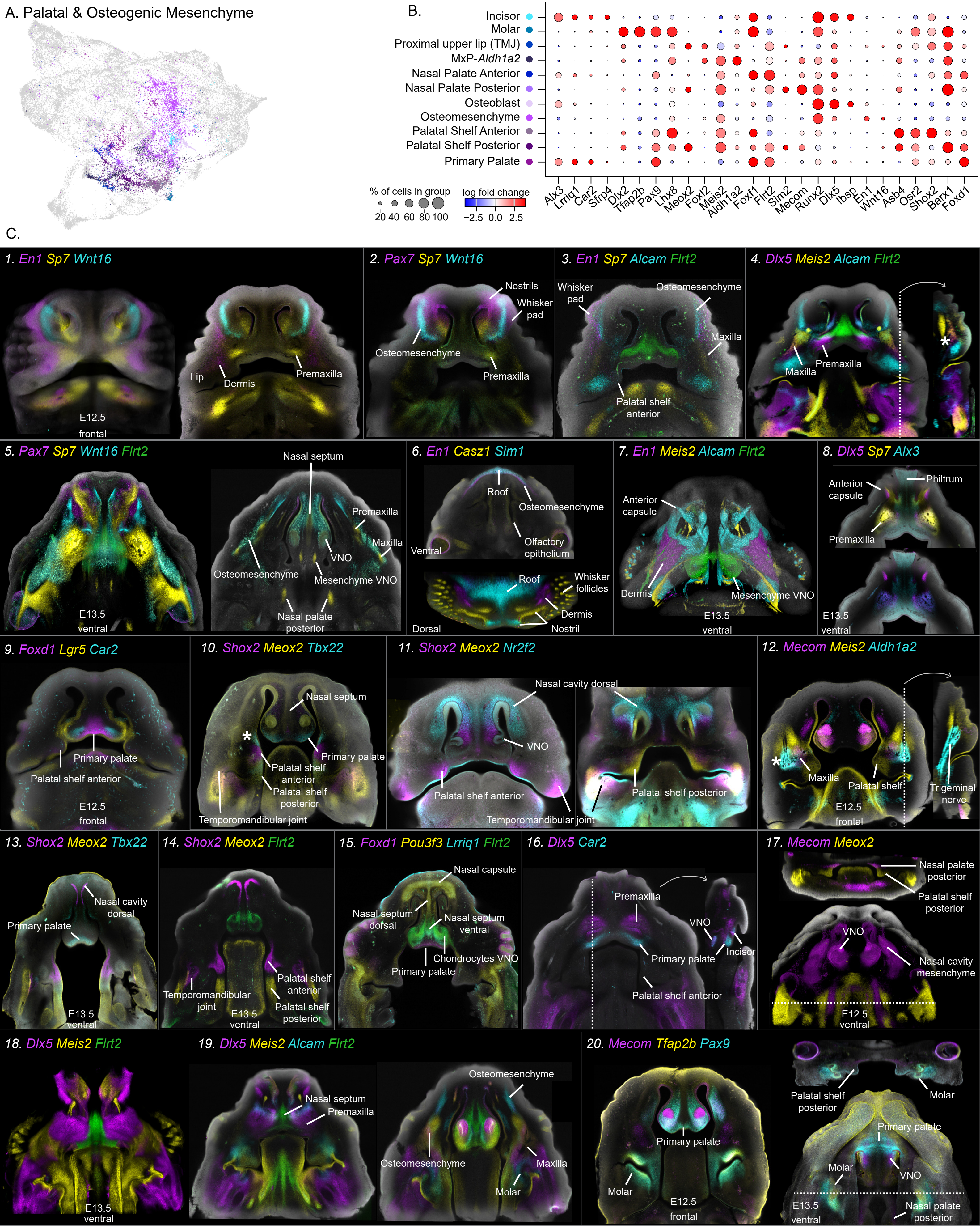

Supplementary Figure 8. Cluster identity and validation for osteogenic and palatal mesenchyme from E12.5 to E14.5.

A) Integrated UMAP highlighting the cells composing the osteogenic, primary palate and palatal shelf mesenchyme, marked by the generalized expression of *Dlx5* and *Runx2*. B) Dot plot of selected genes used for cluster identification, including early positional and canonical cell-type markers. "Proximal upper lip" cluster corresponds to the temporomandibular joint (TMJ). C) Multiplex *in situ* hybridization (HCR) indicating the expression patterns of selected marker genes and the location of clusters listed in (B), in whole mount and cross sections. Vertical broken lines in C1, C12 and C16, and horizontal broken lines in C17 and C21, mark the levels of the sections shown in the corresponding panels. The asterisk in C4 points to the colocalization of *Dlx5* and *Meis2* in the maxillary mesenchyme. The asterisk in C10 shows the fusion point of the primary palate and the anterior palatal shelf. The asterisk in C12 points to the MxP-*Aldh1a*2-positive mesenchyme surrounding the trigeminal nerve.

VNO, vomeronasal organ.

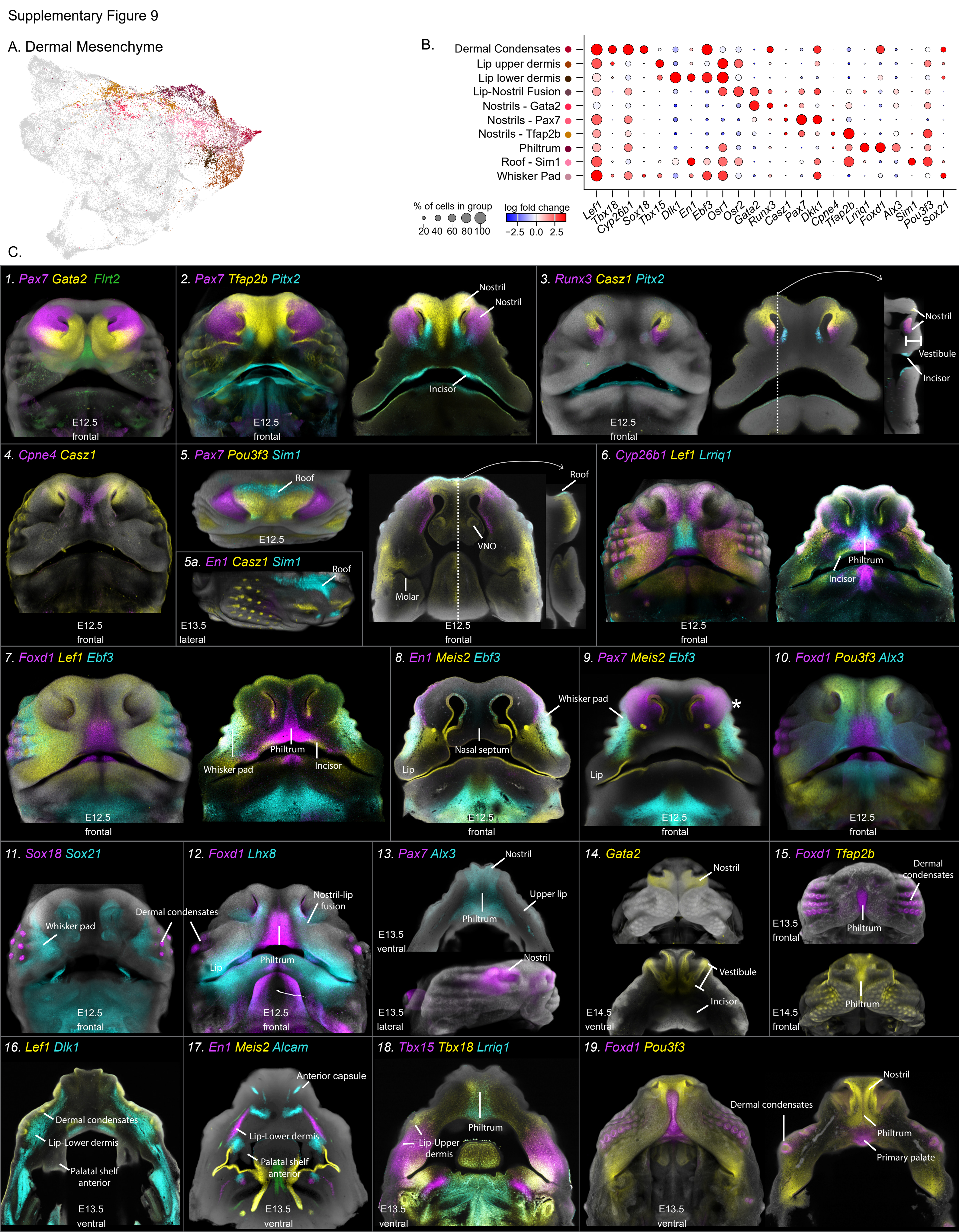

Supplementary Figure 9
A. Dermal Mesenchyme
B.
Dermal Condensates
Lip upper dermis
Lip lower dermis
Lip-Nostril Fusion
Nostrils - Gata2
Nostrils - Pax7
Nostrils - Tfap2b
Philtrum
Roof - Sim1
Whisker Pad
% of cells in group
20 40 60 80 100
log fold change
−2.5 0.0 2.5
Lef1 Tbx18 Cyp26b1 Sox18 Tbx15 Dlk1 En1 Ebf3 Osr1 Osr2 Gata2 Runx3 Casz1 Pax7 Dkk1 Cpne4 Tfap2b Lrriq1 Foxd1 Alx3 Sim1 Pou3f3 Sox21
C.
1. Pax7 Gata2 Flrt2
E12.5 frontal
2. Pax7 Tfap2b Pitx2
Nostril
Nostril
Incisor
E12.5 frontal
3. Runx3 Casz1 Pitx2
Nostril
Vestibule
Incisor
E12.5 frontal
4. Cpne4 Casz1
E12.5 frontal
5. Pax7 Pou3f3 Sim1
Roof
E12.5
Roof
VNO
Molar
E12.5 frontal
5a. En1 Casz1 Sim1
Roof
E13.5 lateral
6. Cyp26b1 Lef1 Lrriq1
Philtrum
Incisor
E12.5 frontal
7. Foxd1 Lef1 Ebf3
Philtrum
Whisker pad
Incisor
E12.5 frontal
8. En1 Meis2 Ebf3
Whisker pad
Nasal septum
Lip
E12.5 frontal
9. Pax7 Meis2 Ebf3
*
Lip
E12.5 frontal
10. Foxd1 Pou3f3 Alx3
E12.5 frontal
11. Sox18 Sox21
Whisker pad
Dermal condensates
E12.5 frontal
12. Foxd1 Lhx8
Nostril-lip fusion
Lip
Philtrum
E12.5 frontal
13. Pax7 Alx3
Nostril
Upper lip
Philtrum
E13.5 ventral
Nostril
E13.5 lateral
14. Gata2
Nostril
Vestibule
Incisor
E14.5 ventral
15. Foxd1 Tfap2b
Dermal condensates
Philtrum
E13.5 frontal
Philtrum
E14.5 frontal
16. Lef1 Dlk1
Dermal condensates
Lip-Lower dermis
Palatal shelf anterior
E13.5 ventral
17. En1 Meis2 Alcam
Anterior capsule
Lip-Lower dermis
Palatal shelf anterior
E13.5 ventral
18. Tbx15 Tbx18 Lrriq1
Philtrum
Lip-Upper dermis
E13.5 ventral
19. Foxd1 Pou3f3
Dermal condensates
Nostril
Philtrum
Primary palate
E13.5 ventral

Supplementary Figure 9. Cluster identity and validation for dermal mesenchyme from E12.5 to E14.5.

A) Integrated UMAP highlighting the cells composing the dermal mesenchyme, marked by the expression of *Lef1*. B) Dot plot of selected genes used for cluster identification, including early positional and canonical cell type markers. C) Multiplex *in situ* hybridization (HCR) indicates the expression patterns of selected marker genes and the location of each dermal cluster in whole mount and cross sections. Broken lines in C3 and C5 mark the levels of the sections shown in the corresponding panels. The asterisk in C9 points to the colocalization of *Pax7* and *Ebf3* in the upper whisker row.

VNO, vomeronasal organ.

Supplementary Figure 10

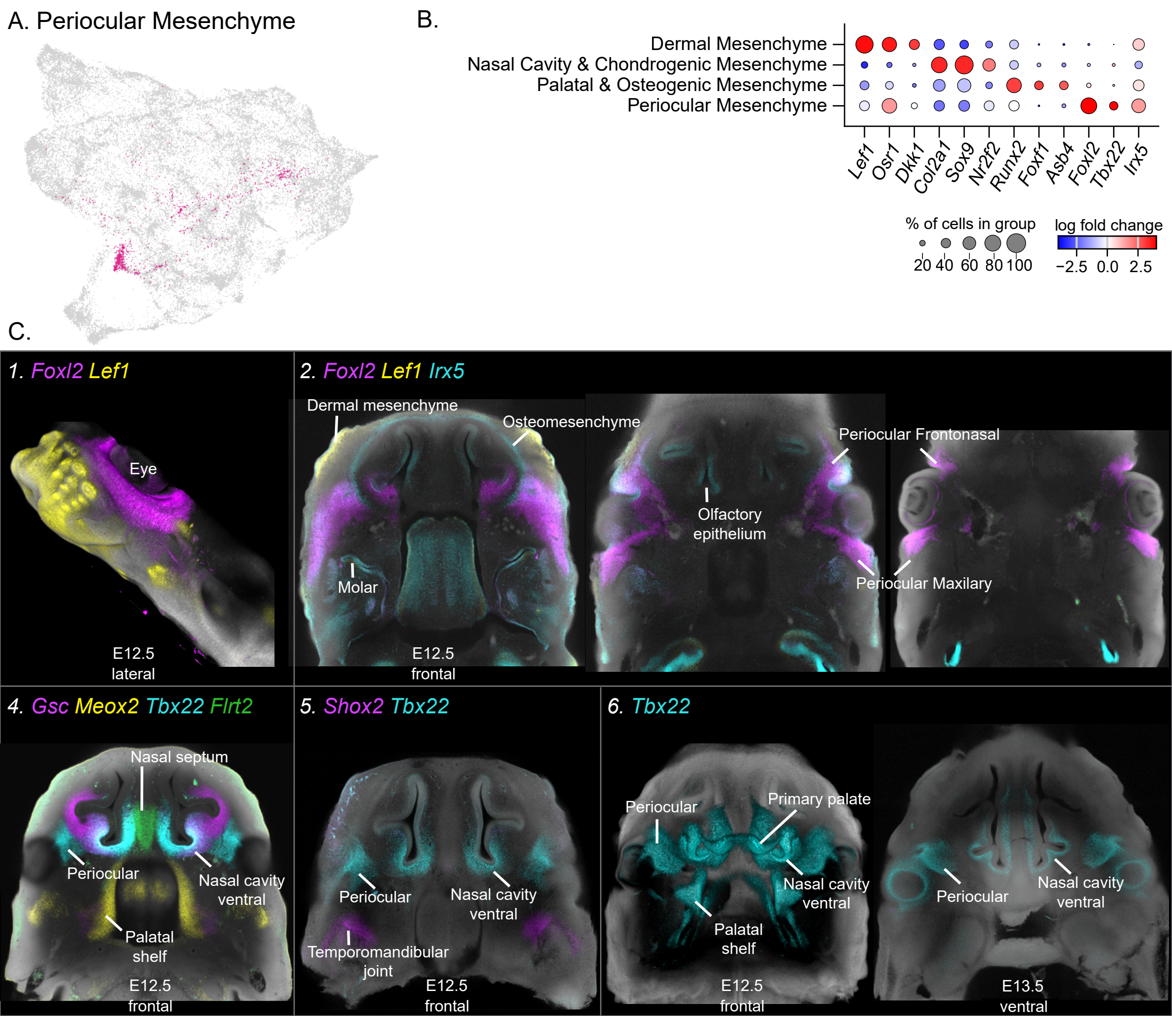

Supplementary Figure 10. Cluster identity and validation for the periocular mesenchyme from E12.5 to E14.5.

A) Integrated UMAP highlighting the cells composing the periocular mesenchyme, marked by the generalized expression of *Foxl2*. B) Dot plot of selected genes used for cluster identification. C) Multiplex *in situ* hybridization (HCR) indicating the expression patterns of selected marker genes and the location of clusters listed in (B), in whole mount and cross sections.

Supplementary Figure 11

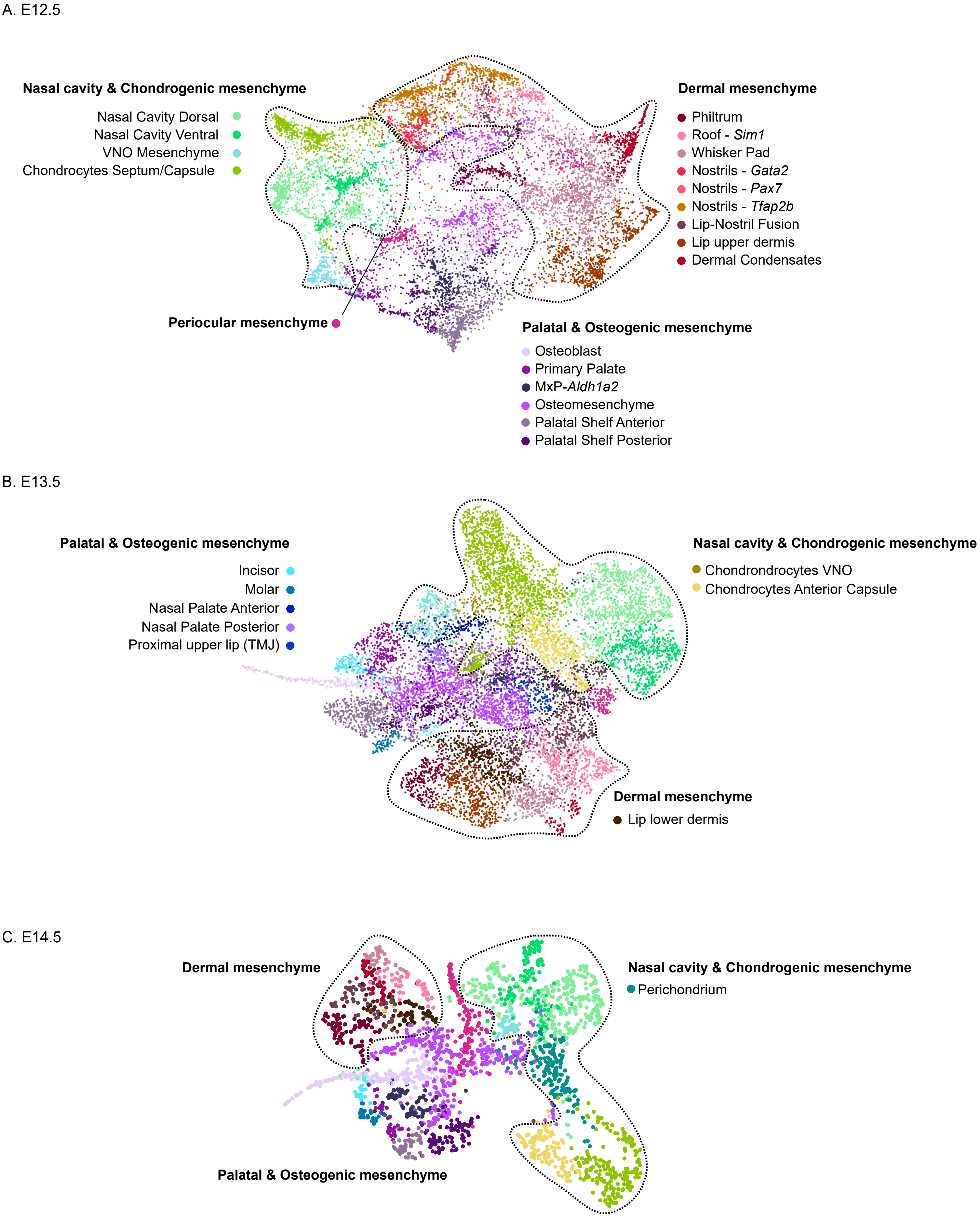

Supplementary Figure 11. Cellular composition and cluster identity of the mesenchyme from E12.5 to E14.5.

A) UMAPs for E12.5, B) E13.5 and C) E14.5, showing the cell cluster composition at each stage. Clusters are grouped into four main cell population nasal cavity and chondrogenic mesenchyme (green shades), palate and osteogenic mesenchyme (purple/blue shades), dermal mesenchyme (red/brown shades) and periocular mesenchyme (magenta). Each UMAP shows all cells present at a given stage. Cluster names listed at each stage correspond to newly emerging cell populations at that stage. Chondrogenic and osteogenic mesenchyme clusters are delineated to aid visualization.

Supplementary Figure 12

Cell communication prediction - All Communication E8.5

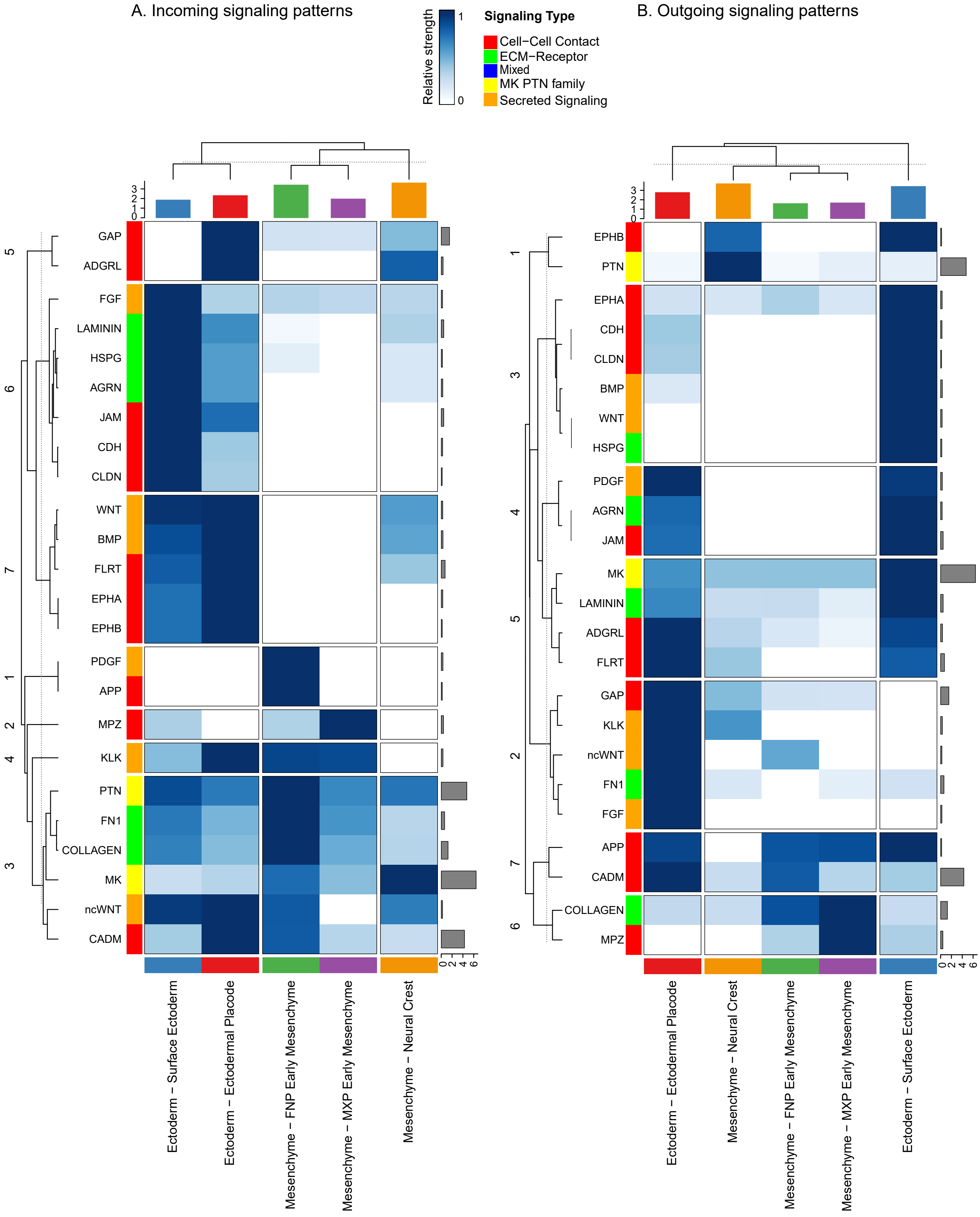

Supplementary Figure 12. Cell-cell communication prediction among ectodermal and mesenchymal populations at E8.5.

Heatmaps displaying relative signaling strength by cluster per pathway, at developmental stage E8.5, split by predicted A) incoming and B) outgoing signaling strength, for the four signaling categories analyzed. "Mixed" denotes signals that fall into more than one signaling category as described by CellChat (http://www.cellchat.org/cellchatdb/). Rows represent signaling pathways, and columns represent clusters. The blue color intensity of each cell indicates the relative signaling strength of the specific pathway in that cluster. Rows and columns were grouped via k-means clustering to highlight the predicted signaling patterns, also displayed by the dendrograms on the left and top of the heatmaps. The numbers on the left dendrogram correspond to the different patterns of signaling pathways identified. The color bars on top of the heatmaps indicate the overall signaling strength contributed by each cluster relative to the total predicted signaling strength. The grey bars on the right represent the overall signaling strength of each pathway compared to the total predicted signaling.

Supplementary Figure 13

Cell communication prediction - All Communication E9.5

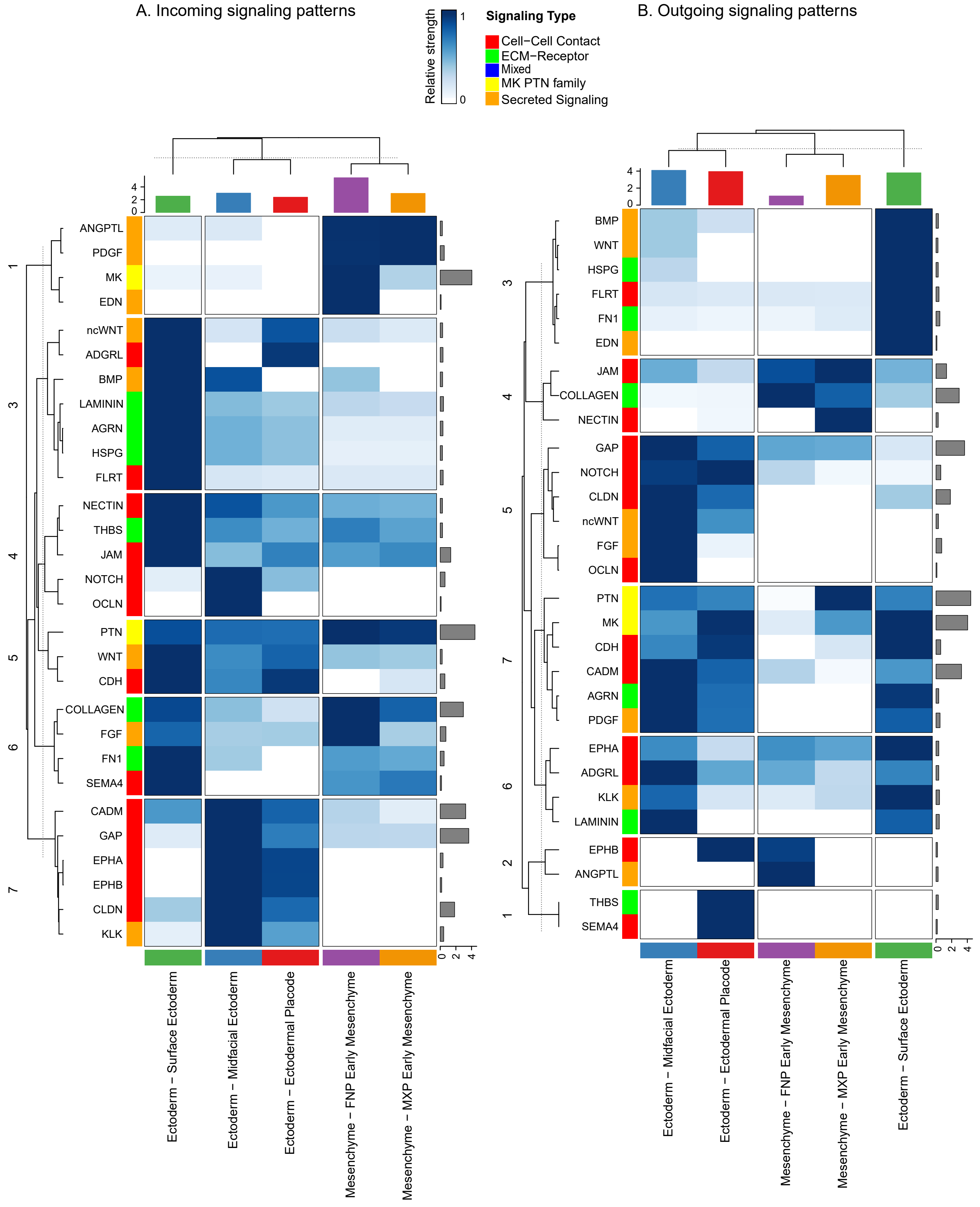

Supplementary Figure 13. Cell-cell communication prediction among ectodermal and mesenchymal populations at E9.5.

Heatmaps displaying relative signaling strength by cluster per pathway, at developmental stage E9.5, split by predicted A) incoming and B) outgoing signaling strength, for the four signaling categories analyzed. "Mixed" denotes signals that fall into more than one signaling category as described by CellChat (http://www.cellchat.org/cellchatdb/). Rows represent signaling pathways, and columns represent clusters. The blue color intensity of each cell indicates the relative signaling strength of the specific pathway in that cluster. Rows and columns were grouped via k-means clustering to highlight the predicted signaling patterns, also displayed by the dendrograms on the left and top of the heatmaps. The numbers on the left dendrogram correspond to the different patterns of signaling pathways identified. The color bars on top of the heatmaps indicate the overall signaling strength contributed by each cluster relative to the total predicted signaling strength. The grey bars on the right represent the overall signaling strength of each pathway compared to the total predicted signaling.

Supplementary Figure 14

Cell communication prediction - All Communication E10.5

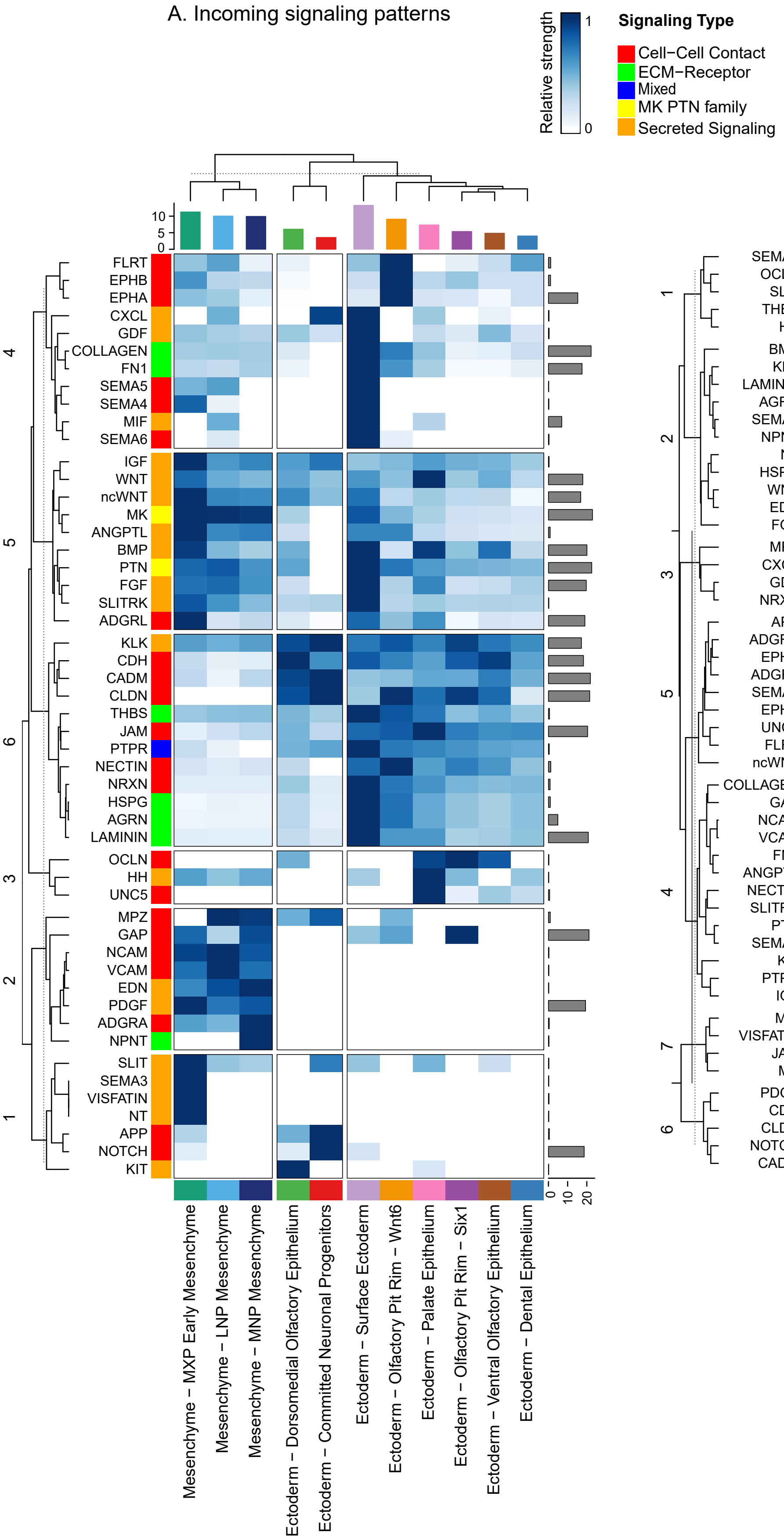

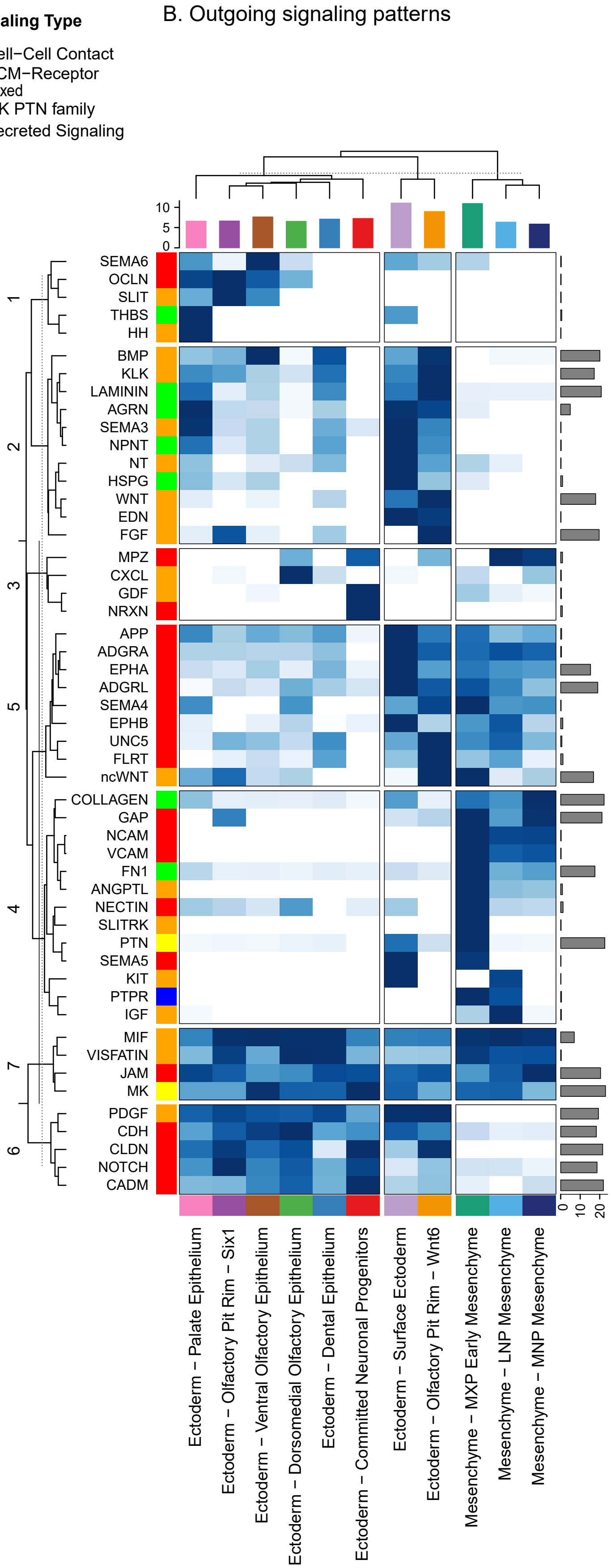

Supplementary Figure 14. Cell-cell communication prediction among ectodermal and mesenchymal populations at E10.5.

Heatmaps displaying relative signaling strength by cluster per pathway, at developmental stage E10.5, split by predicted A) incoming and B) outgoing signaling strength, for the four signaling categories analyzed. "Mixed" denotes signals that fall into more than one signaling category as described by CellChat (http://www.cellchat.org/cellchatdb/). Rows represent signaling pathways, and columns represent clusters. The blue color intensity of each cell indicates the relative signaling strength of the specific pathway in that cluster. Rows and columns were grouped via k-means clustering to highlight the predicted signaling patterns, also displayed by the dendrograms on the left and top of the heatmaps. The numbers on the left dendrogram correspond to the different patterns of signaling pathways identified. The color bars on top of the heatmaps indicate the overall signaling strength contributed by each cluster relative to the total predicted signaling strength. The grey bars on the right represent the overall signaling strength of each pathway compared to the total predicted signaling.

Supplementary Figure 15

Cell communication prediction - All Communication E11.5

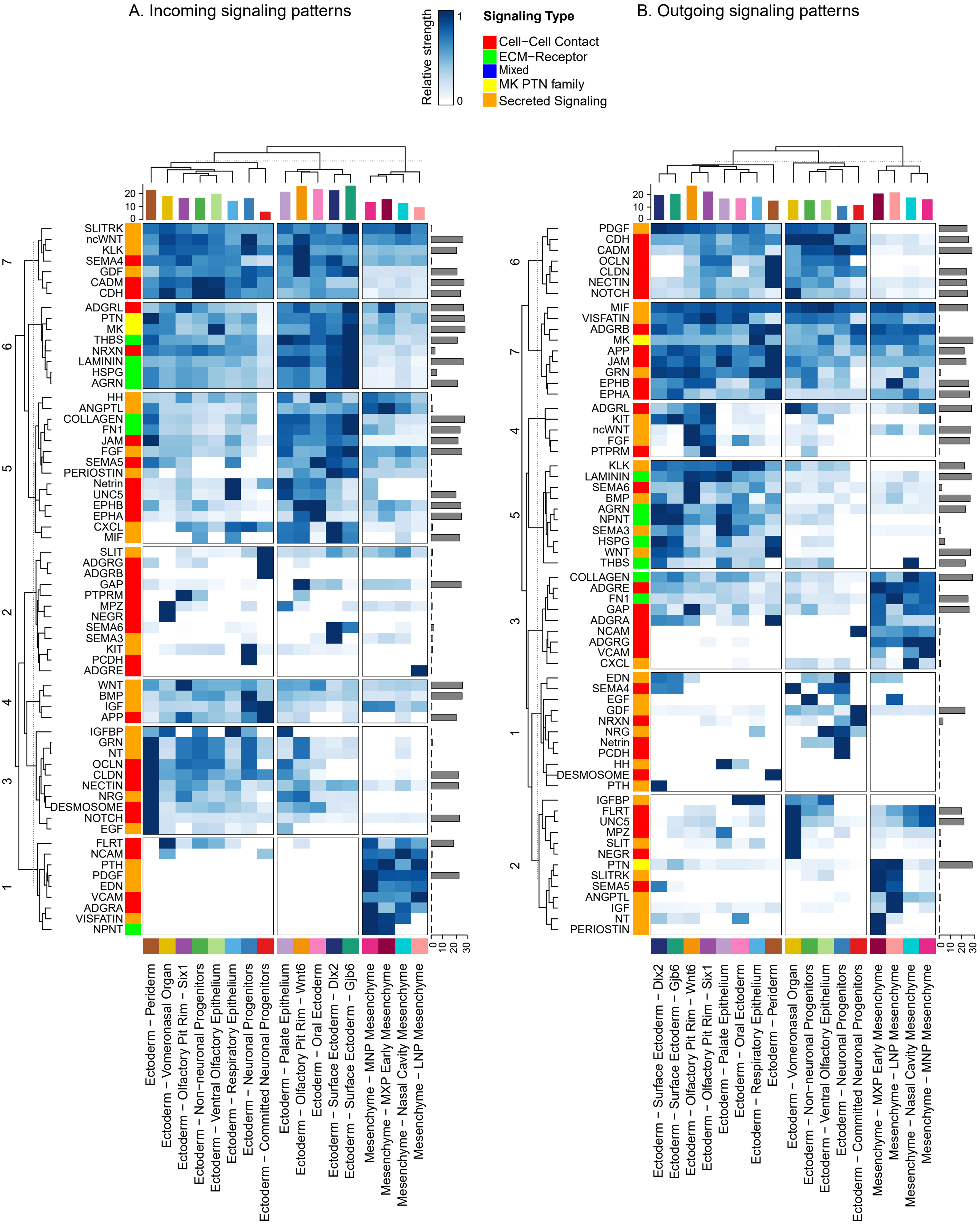

Supplementary Figure 15. Cell-cell communication prediction among ectodermal and mesenchymal populations at E11.5.

Heatmaps displaying relative signaling strength by cluster per pathway, at developmental stage E11.5, split by predicted A) incoming and B) outgoing signaling strength, for the four signaling categories analyzed. "Mixed" denotes signals that fall into more than one signaling category as described by CellChat (http://www.cellchat.org/cellchatdb/). Rows represent signaling pathways, and columns represent clusters. The blue color intensity of each cell indicates the relative signaling strength of the specific pathway in that cluster. Rows and columns were grouped via k-means clustering to highlight the predicted signaling patterns, also displayed by the dendrograms on the left and top of the heatmaps. The numbers on the left dendrogram correspond to the different patterns of signaling pathways identified. The color bars on top of the heatmaps indicate the overall signaling strength contributed by each cluster relative to the total predicted signaling strength. The grey bars on the right represent the overall signaling strength of each pathway compared to the total predicted signaling.

Supplementary Figure 16

Cell communication prediction - All Communication E12.5

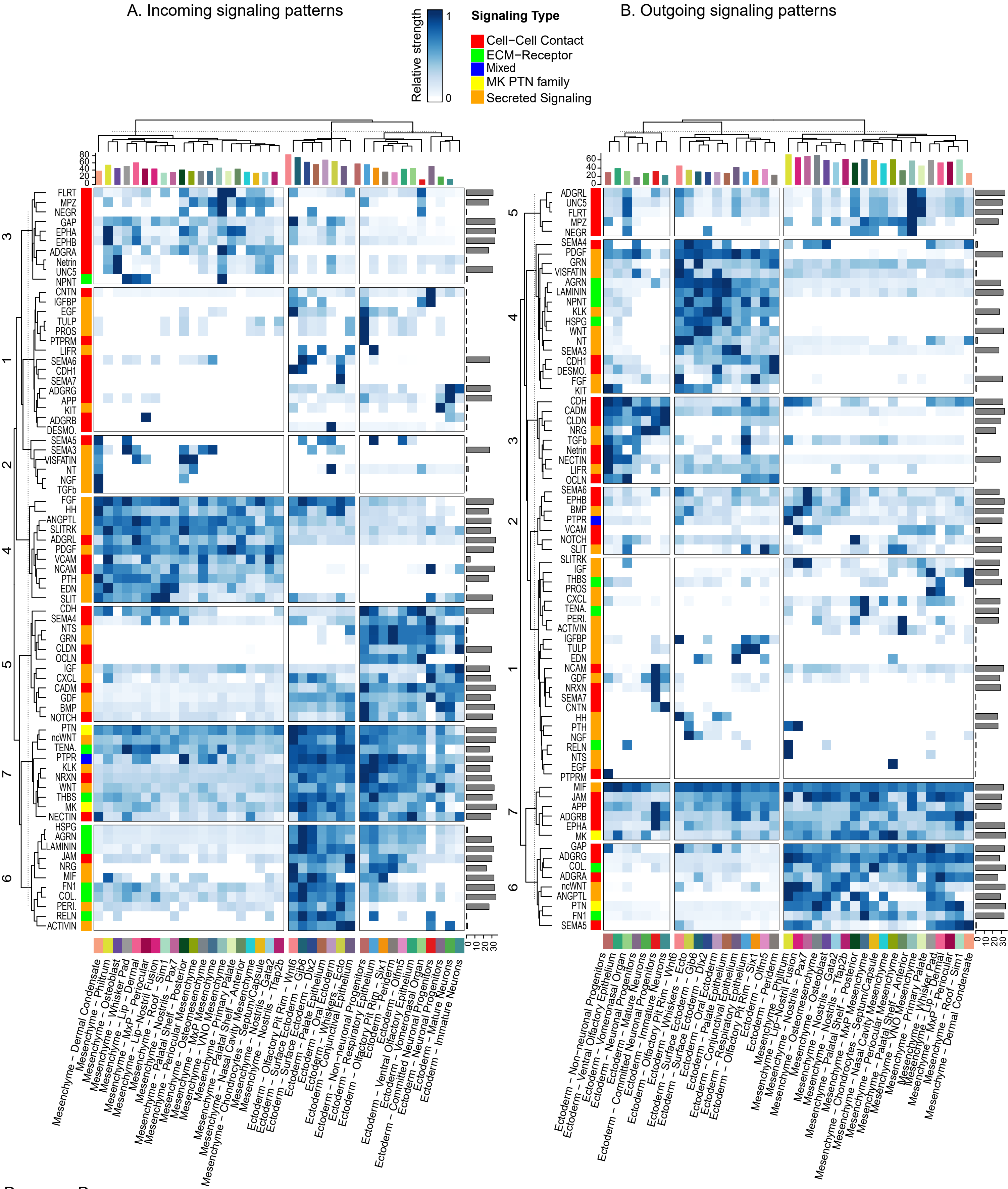

Desmo. = Desmosome
Tena.= Tenascin
Coll. = Collagen
Peri.= Periostin

Supplementary Figure 16. Cell-cell communication prediction among ectodermal and mesenchymal populations at E12.5.

Heatmaps displaying relative signaling strength by cluster per pathway, at developmental stage E12.5, split by predicted A) incoming and B) outgoing signaling strength, for the four signaling categories analyzed. "Mixed" denotes signals that fall into more than one signaling category as described by CellChat (http://www.cellchat.org/cellchatdb/). Rows represent signaling pathways, and columns represent clusters. The blue color intensity of each cell indicates the relative signaling strength of the specific pathway in that cluster. Rows and columns were grouped via k-means clustering to highlight the predicted signaling patterns, also displayed by the dendrograms on the left and top of the heatmaps. The numbers on the left dendrogram correspond to the different patterns of signaling pathways identified. The color bars on top of the heatmaps indicate the overall signaling strength contributed by each cluster relative to the total predicted signaling strength. The grey bars on the right represent the overall signaling strength of each pathway compared to the total predicted signaling.

Supplementary Figure 17

Cell communication prediction - All Communication E13.5

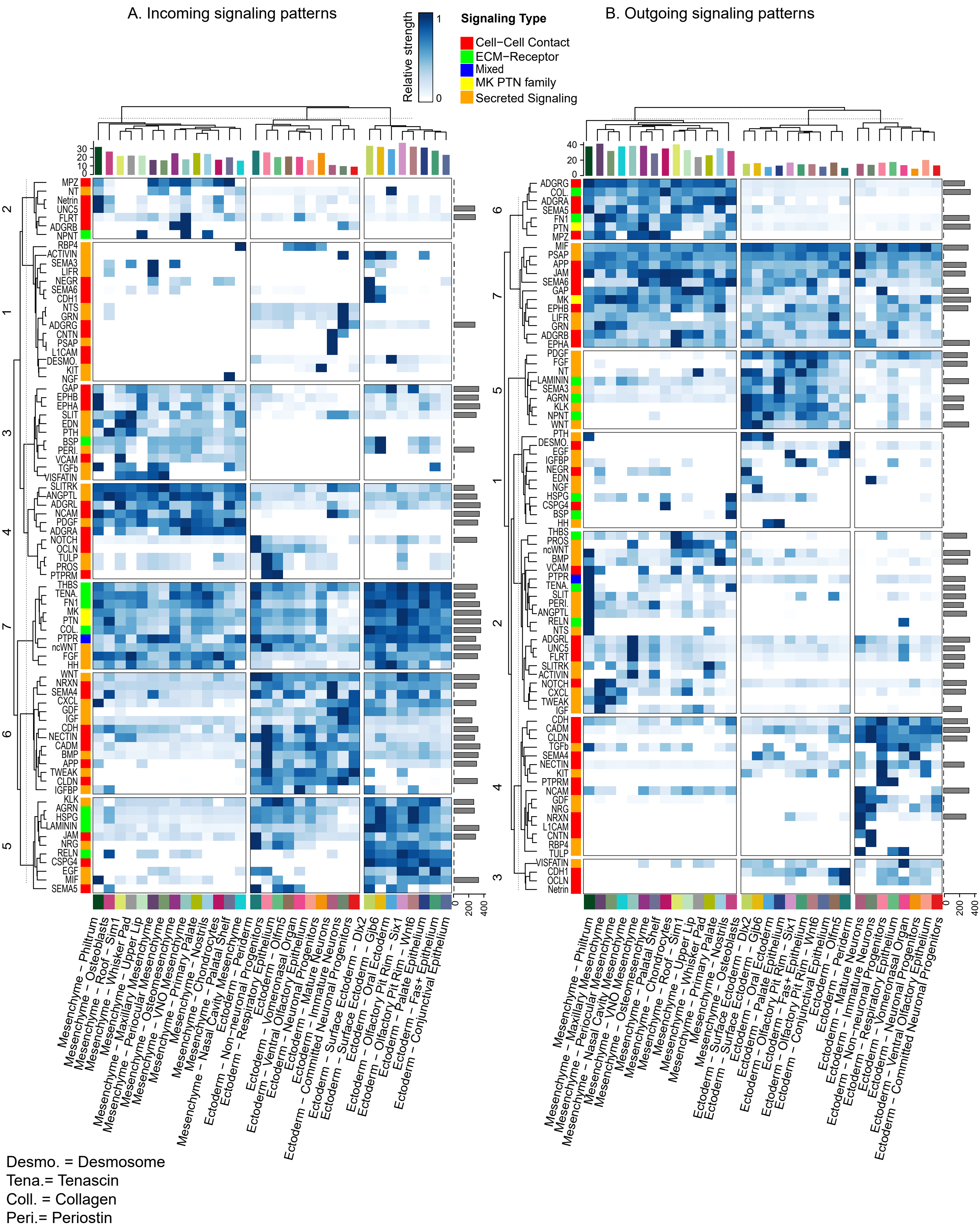

Desmo. = Desmosome
Tena.= Tenascin
Coll. = Collagen
Peri.= Periostin

Supplementary Figure 17. Cell-cell communication prediction among ectodermal and mesenchymal populations at E13.5.

Heatmaps displaying relative signaling strength by cluster per pathway, at developmental stage E13.5, split by predicted A) incoming and B) outgoing signaling strength, for the four signaling categories analyzed. "Mixed" denotes signals that fall into more than one signaling category as described by CellChat (http://www.cellchat.org/cellchatdb/). Rows represent signaling pathways, and columns represent clusters. The blue color intensity of each cell indicates the relative signaling strength of the specific pathway in that cluster. Rows and columns were grouped via k-means clustering to highlight the predicted signaling patterns, also displayed by the dendrograms on the left and top of the heatmaps. The numbers on the left dendrogram correspond to the different patterns of signaling pathways identified. The color bars on top of the heatmaps indicate the overall signaling strength contributed by each cluster relative to the total predicted signaling strength. The grey bars on the right represent the overall signaling strength of each pathway compared to the total predicted signaling.

Supplementary Figure 18

Cell communication prediction - All Communication E14.5

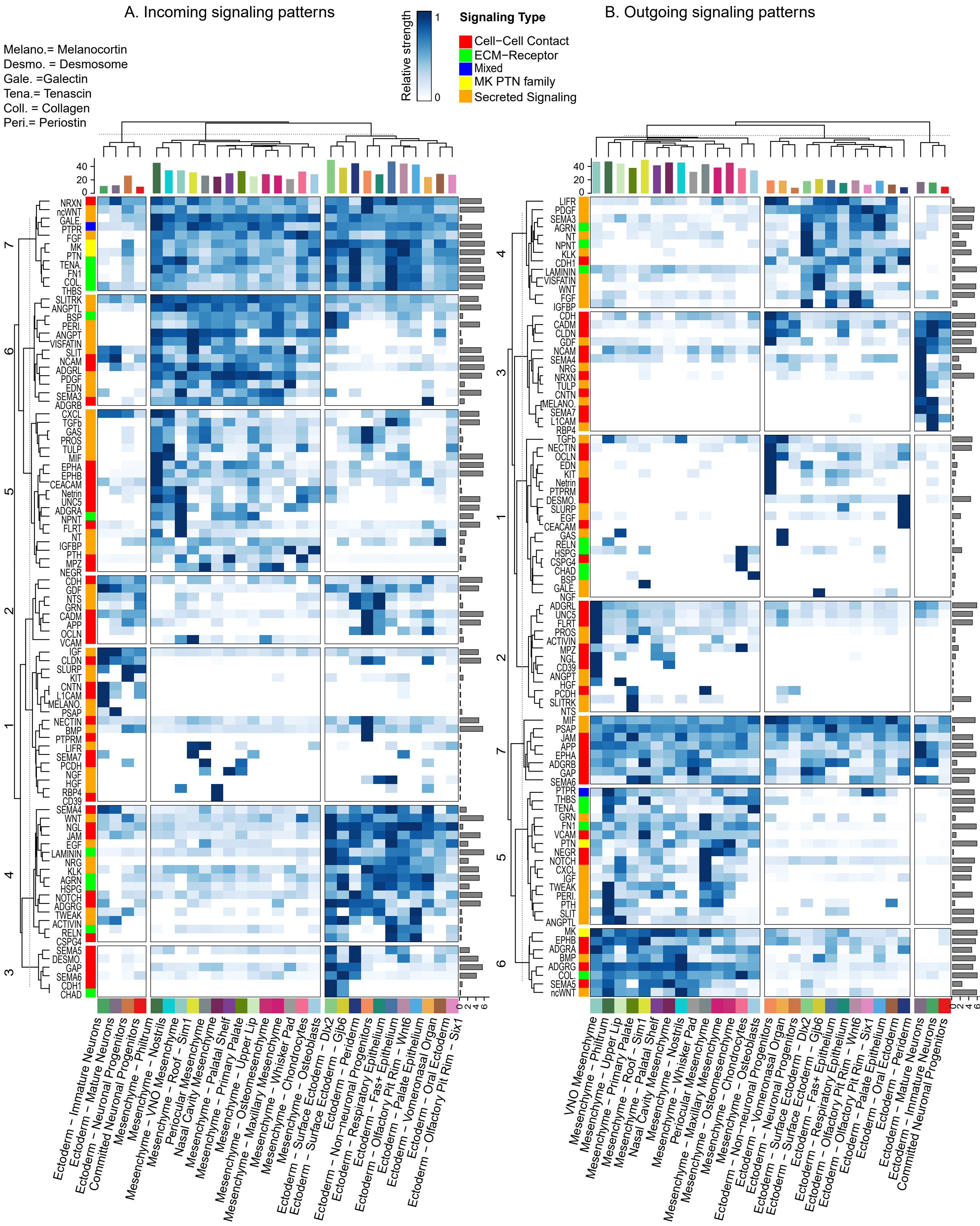

Supplementary Figure 18. Cell-cell communication prediction among ectodermal and mesenchymal populations at E14.5.

Heatmaps displaying relative signaling strength by cluster per pathway, at developmental stage E14.5, split by predicted A) incoming and B) outgoing signaling strength, for the four signaling categories analyzed. "Mixed" denotes signals that fall into more than one signaling category as described by CellChat (http://www.cellchat.org/cellchatdb/). Rows represent signaling pathways, and columns represent clusters. The blue color intensity of each cell indicates the relative signaling strength of the specific pathway in that cluster. Rows and columns were grouped via k-means clustering to highlight the predicted signaling patterns, also displayed by the dendrograms on the left and top of the heatmaps. The numbers on the left dendrogram correspond to the different patterns of signaling pathways identified. The color bars on top of the heatmaps indicate the overall signaling strength contributed by each cluster relative to the total predicted signaling strength. The grey bars on the right represent the overall signaling strength of each pathway compared to the total predicted signaling.

Supplementary Figure 19 - Part 1

Predicted incoming and outgoing aggregated signaling probability

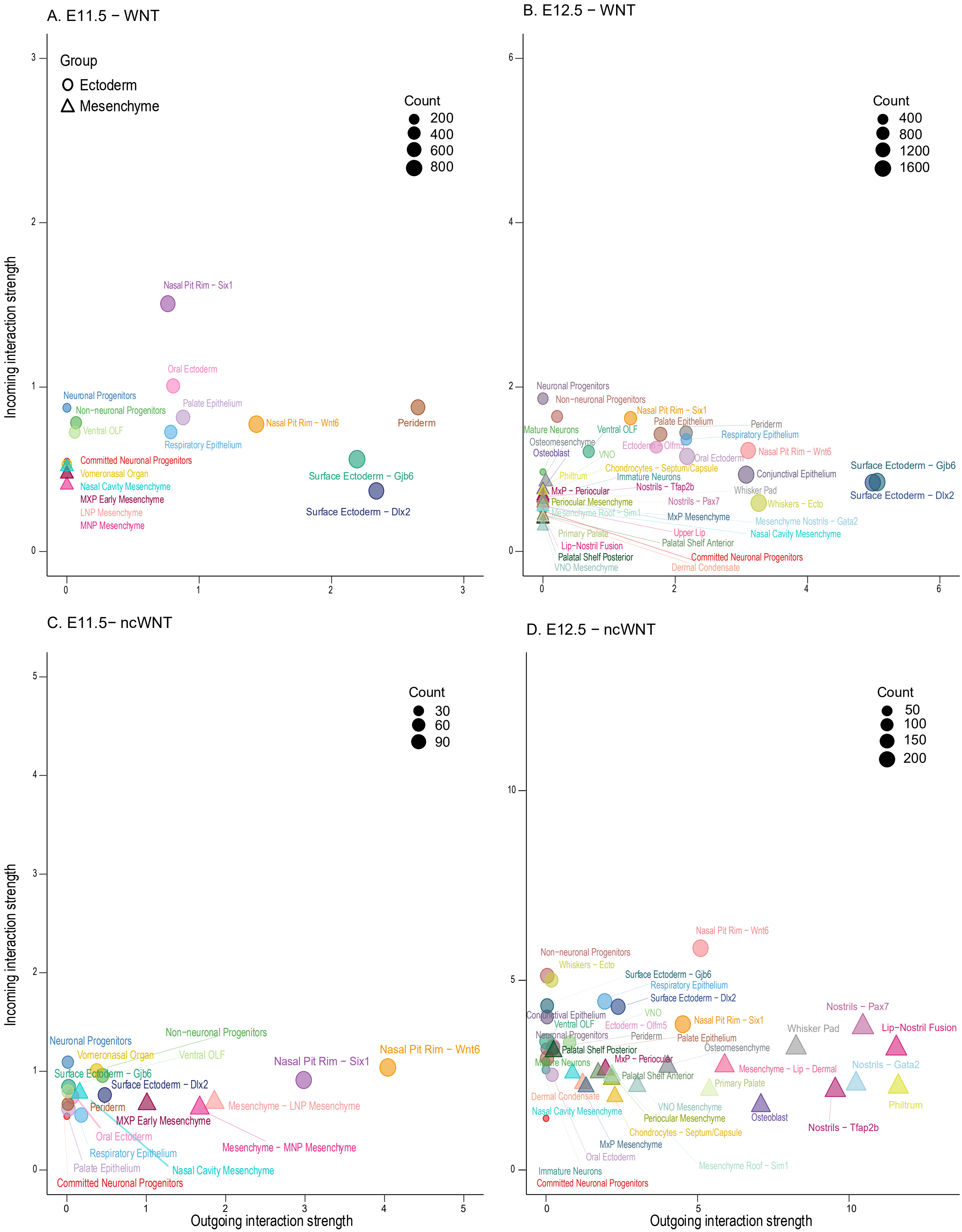

Supplementary Figure 19 - Part 2

Predicted incoming and outgoing aggregated signaling probability

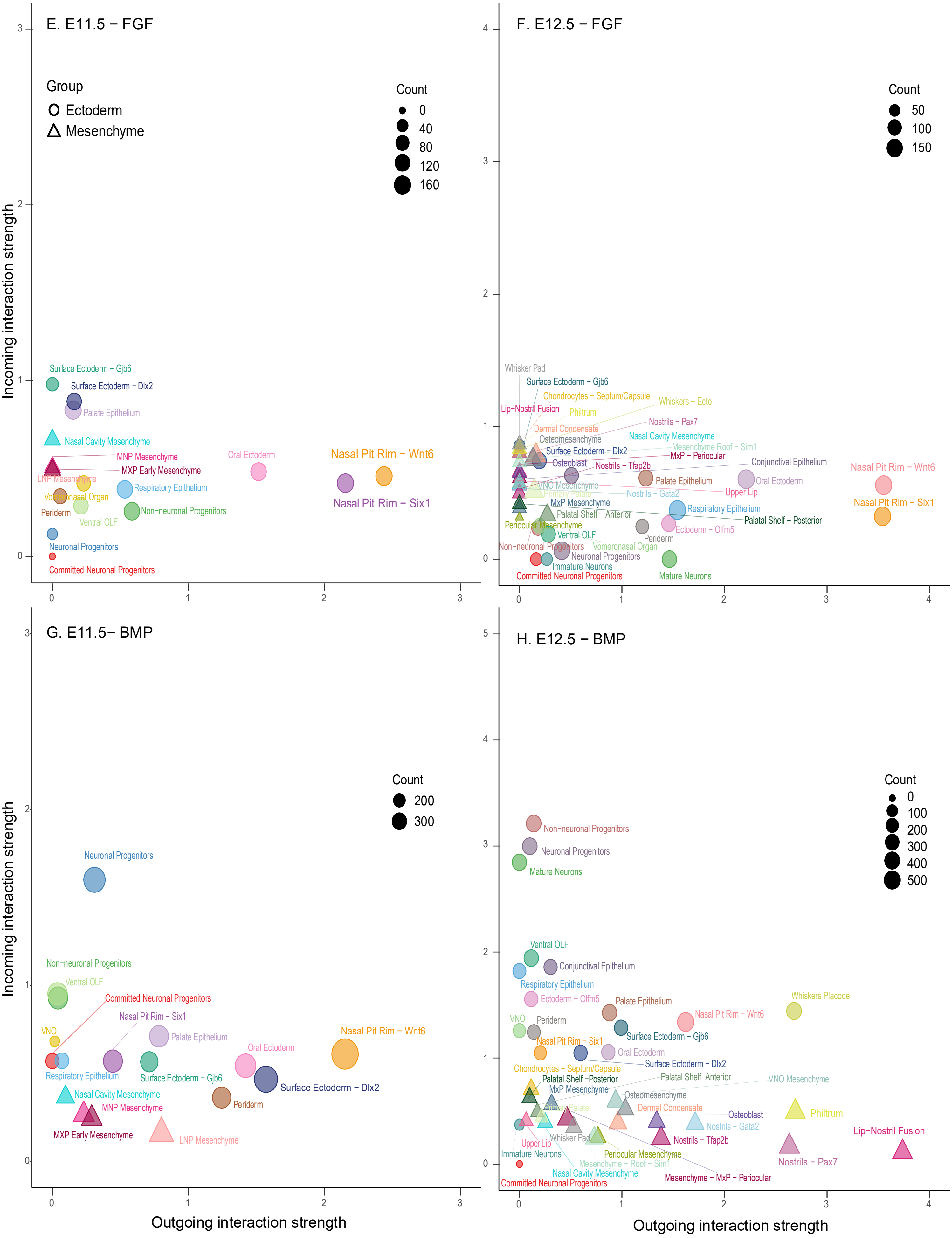

Supplementary Figure 19 - Part 3

Predicted incoming and outgoing aggregated signaling probability

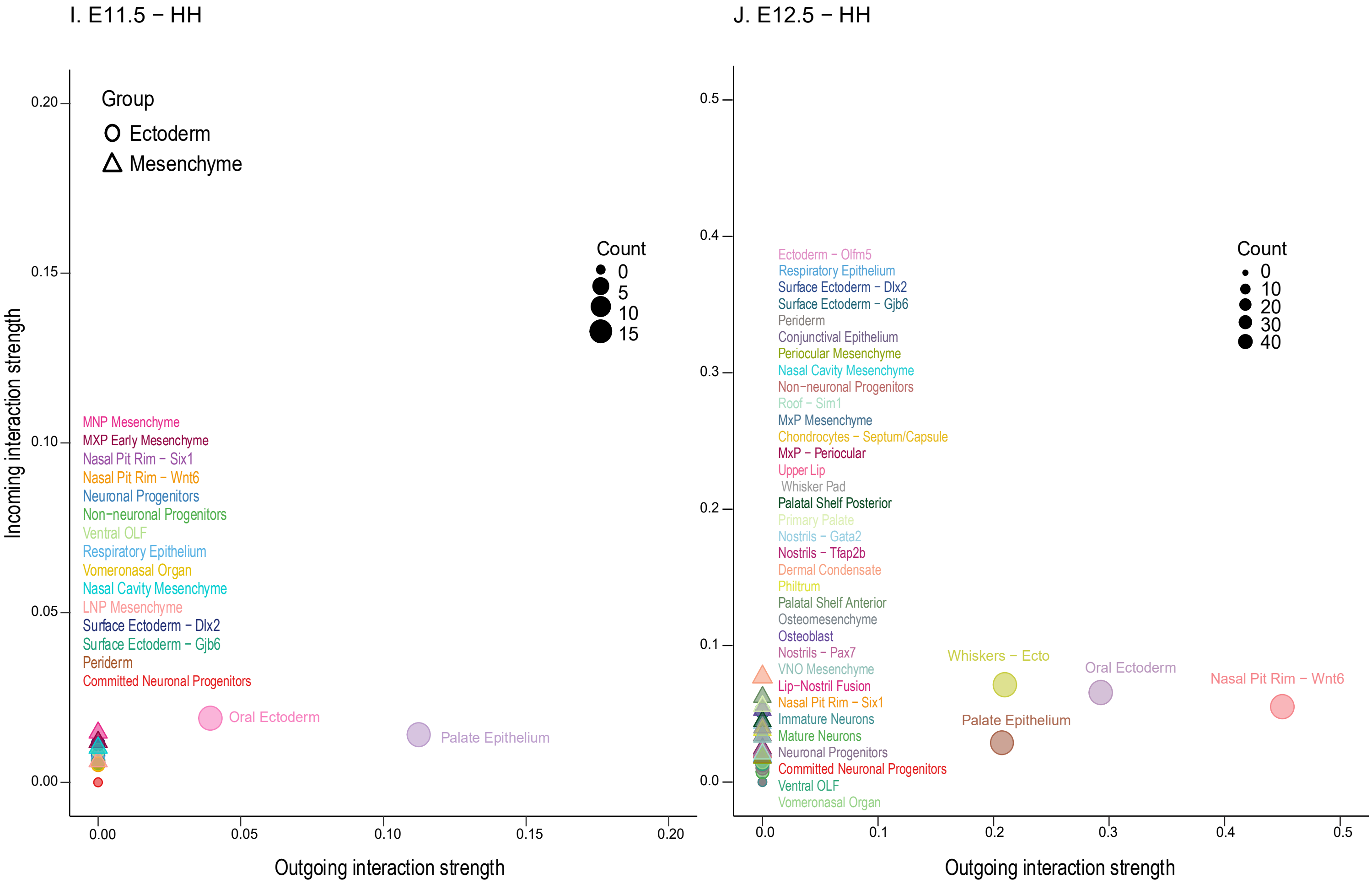

Supplementary Figure 19. Signaling role analysis on the aggregated cell-cell communication network from selected signaling pathways.

Scatter plots of predicted incoming and outgoing aggregated secreted signaling probabilities per cluster of selected signaling pathways, at E11.5 (A, C, E, G, I) and E12.5 (B, D, F, H, J). The x- and y-axes represent the total incoming or outgoing signaling probability, respectively. Circles and triangles indicate ectodermal and mesenchymal clusters, respectively. Shape colors represent different clusters. Shape size is proportional to the number of predicted signaling links (both outgoing and incoming) associated with each cluster.

OLF, Olfactory epithelium; VNO, Vomeronasal organ.

Supplementary Figure 20

A. Predicted molecular communication

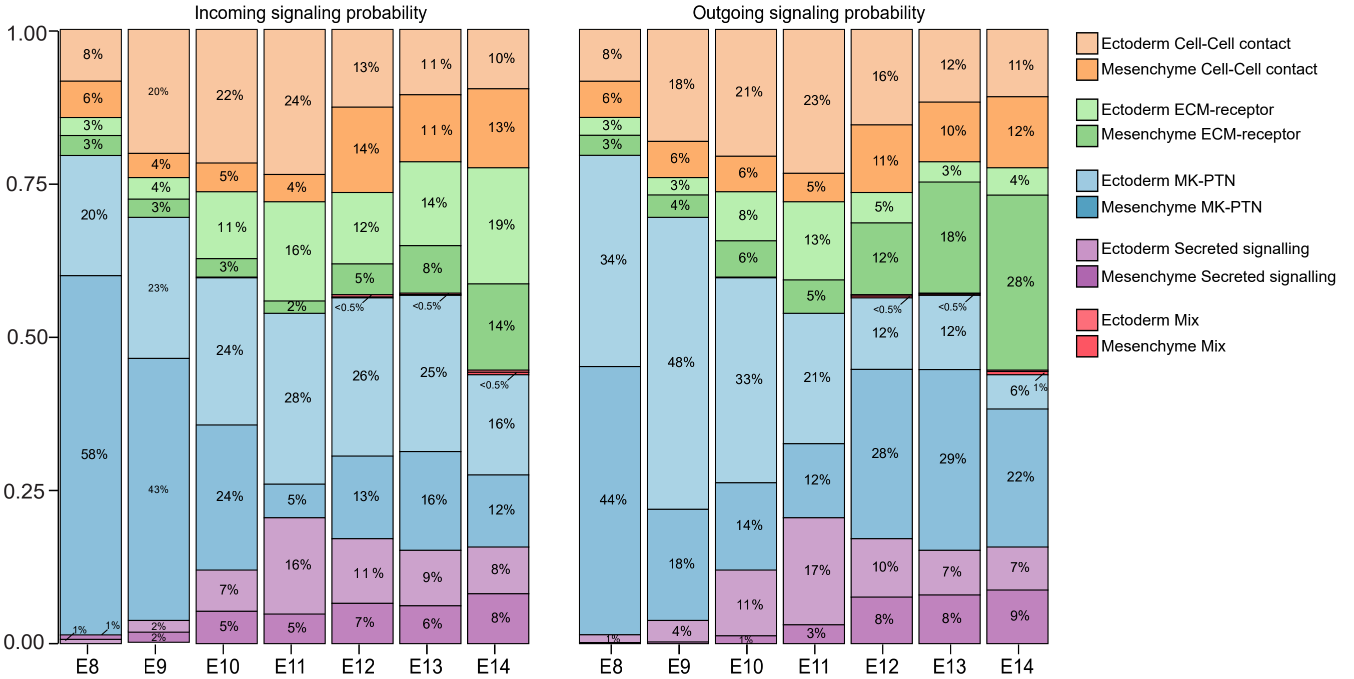

B. Predicted molecular communication without MK-PTN signalling

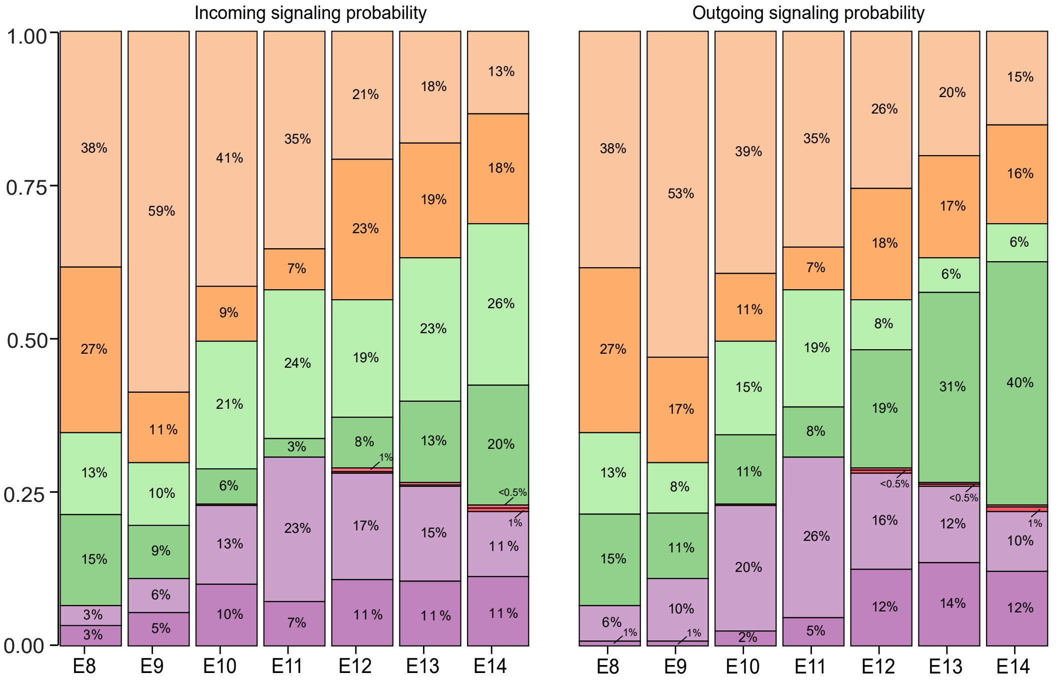

Supplementary Figure 20. Predicted cellular communication between ectodermal and mesenchymal populations.

A-B) Stacked bar plots showing relative signaling probability (i.e., predicted likelihood of interaction via a given pathway) for the four signaling categories (A) and excluding MK/PTN pathways (B), analyzed per stage. Incoming (left) and outgoing (right) signaling between ectoderm and mesenchyme are shown separately. Colors represent signaling categories; light and dark shades distinguish ectoderm and mesenchyme. Percentages indicate the relative contribution of each signaling category.

Supplementary Figure 21

Enrichment patterns of GWAS genes (EFO 0007841), associated with human normal facial shape variation

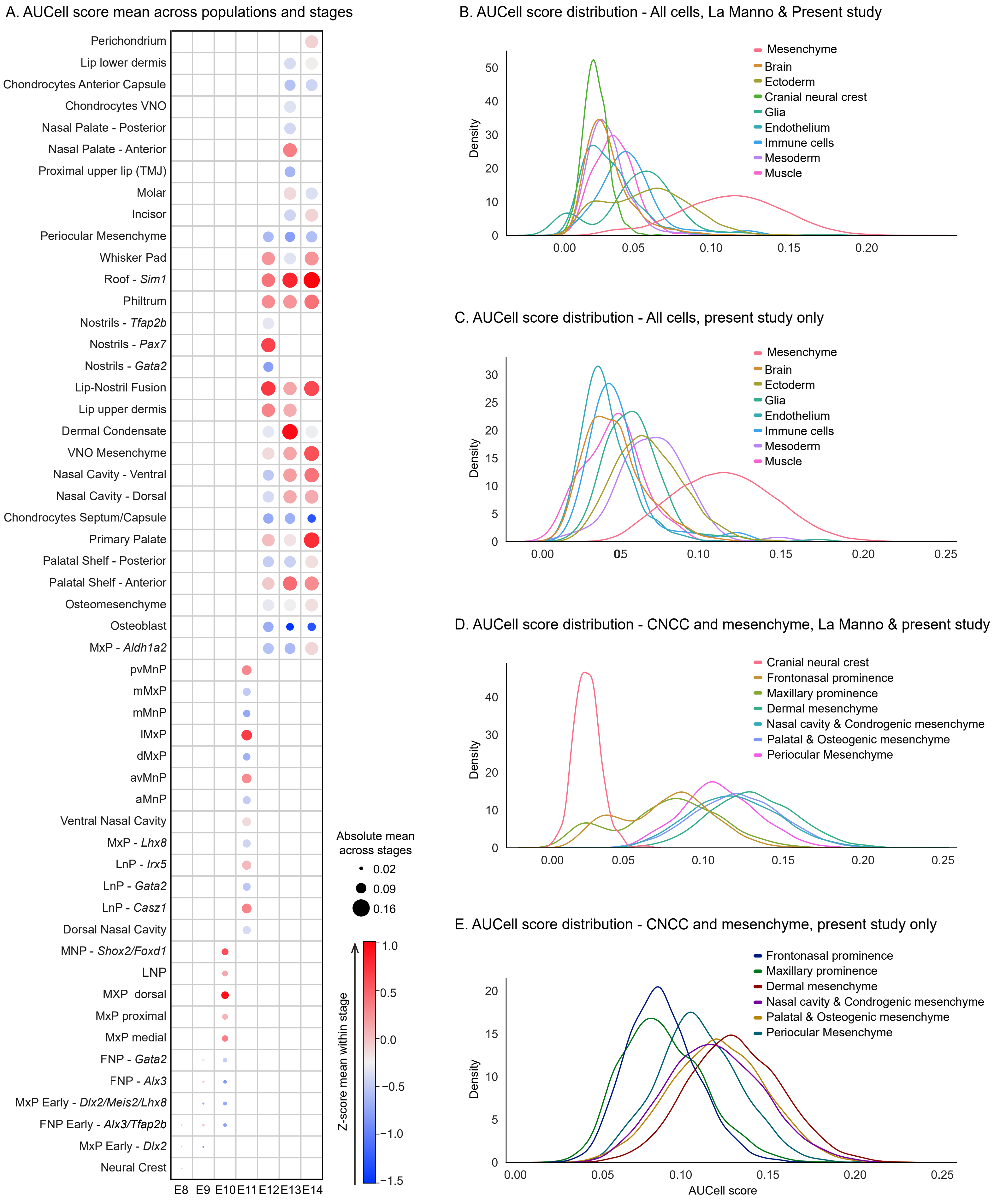

Supplementary Figure 21. Enrichment patterns of GWAS gene set associated with human normal facial shape variation.

A) Dot plot of AUCell scores based on the normal shape variation GWAS gene set (Supp. Data 5) across mesenchymal populations and developmental stages. Columns correspond to developmental stages and rows to cell populations (*i.e.* clusters). The size of the dot represents the mean AUCell score in that population at that developmental stage. The color of the dot indicates the stage-wise Z-score (standard deviation to the mean), highlighting enrichment differences among populations within a given stage (*e.g.* the redder the circle, the greater the enrichment of the gene set in that population compared to others in the same stage). It is important to note the size of the dot when comparing populations across stages. B) Density curve plot showing the AUCell score distribution between cell types, using both datasets (La Manno and present study). C) Similar to (B), using only the cells from present study dataset, ruling out any batch effect in the density distribution pattern. D-E), similar to (B) and (C), respectively, showing the distribution of AUCell scores in the cranial neural crest cells (CNCCs) and mesenchyme subset.

Supplementary Figure 22

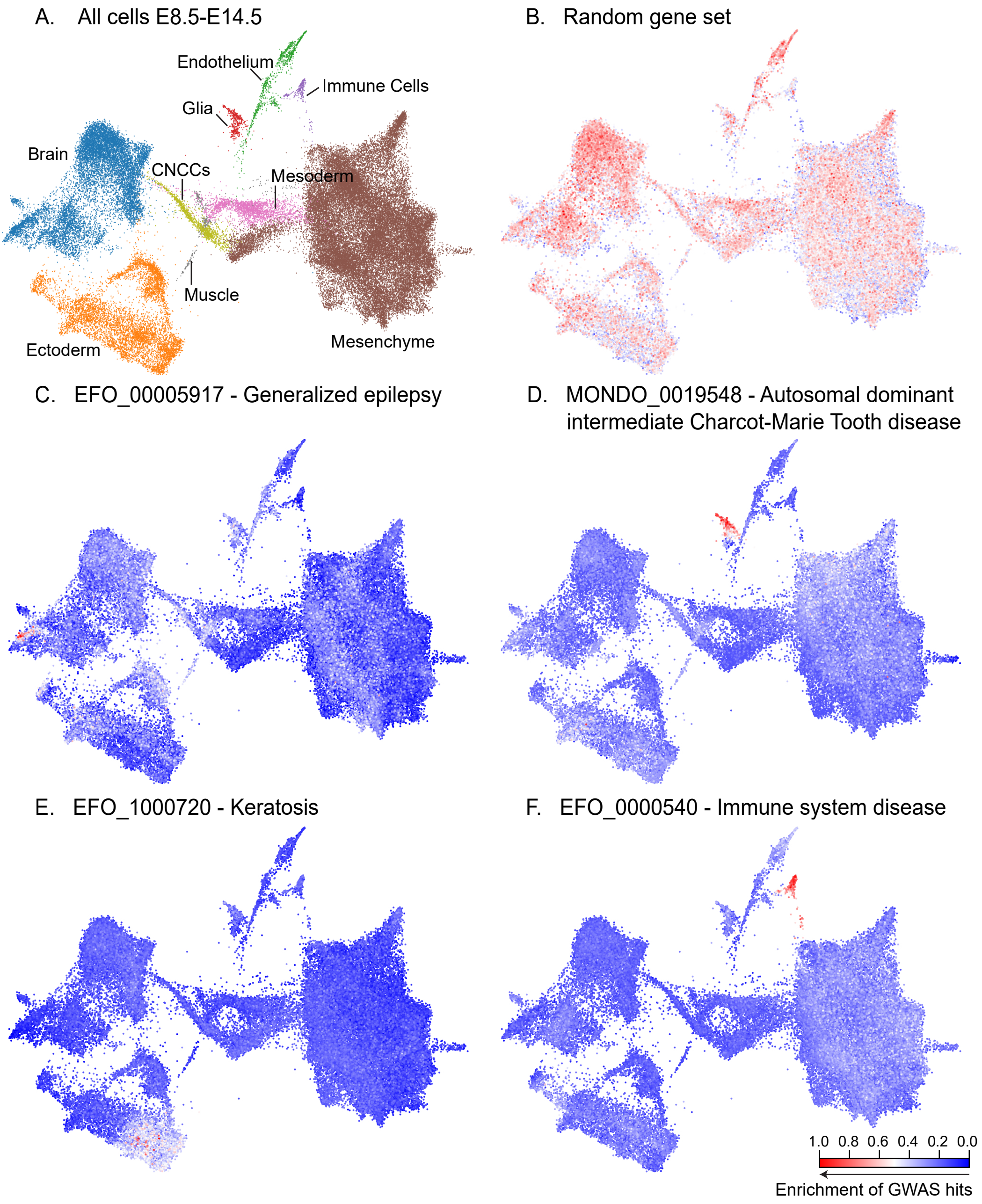

Supplementary Figure 22. AUCell analysis control using known tissue-specific phenotypes.

A) Integrated UMAP for all cells analyzed (E8.5-E14.5) as in Fig. 1B. B-F) Relative AUCell scores for genes associated with a random set of genes (B), EFO_00005917-Generalized epilepsy (C), MONDO_0019548-Autosomal dominant intermediate Charcot-Marie Tooth disease (D), EFO_1000720 - Keratosis (E), and EFO_0000540-Immune system disease (F). Note random enrichment in (B) and specific enrichment in brain (C), glia (D), surface ectoderm (E) and immune cells (F) clusters. These gene sets are presented in Supp. Data 7.

Supplementary Figure 23

Pax3 expression during mouse face development

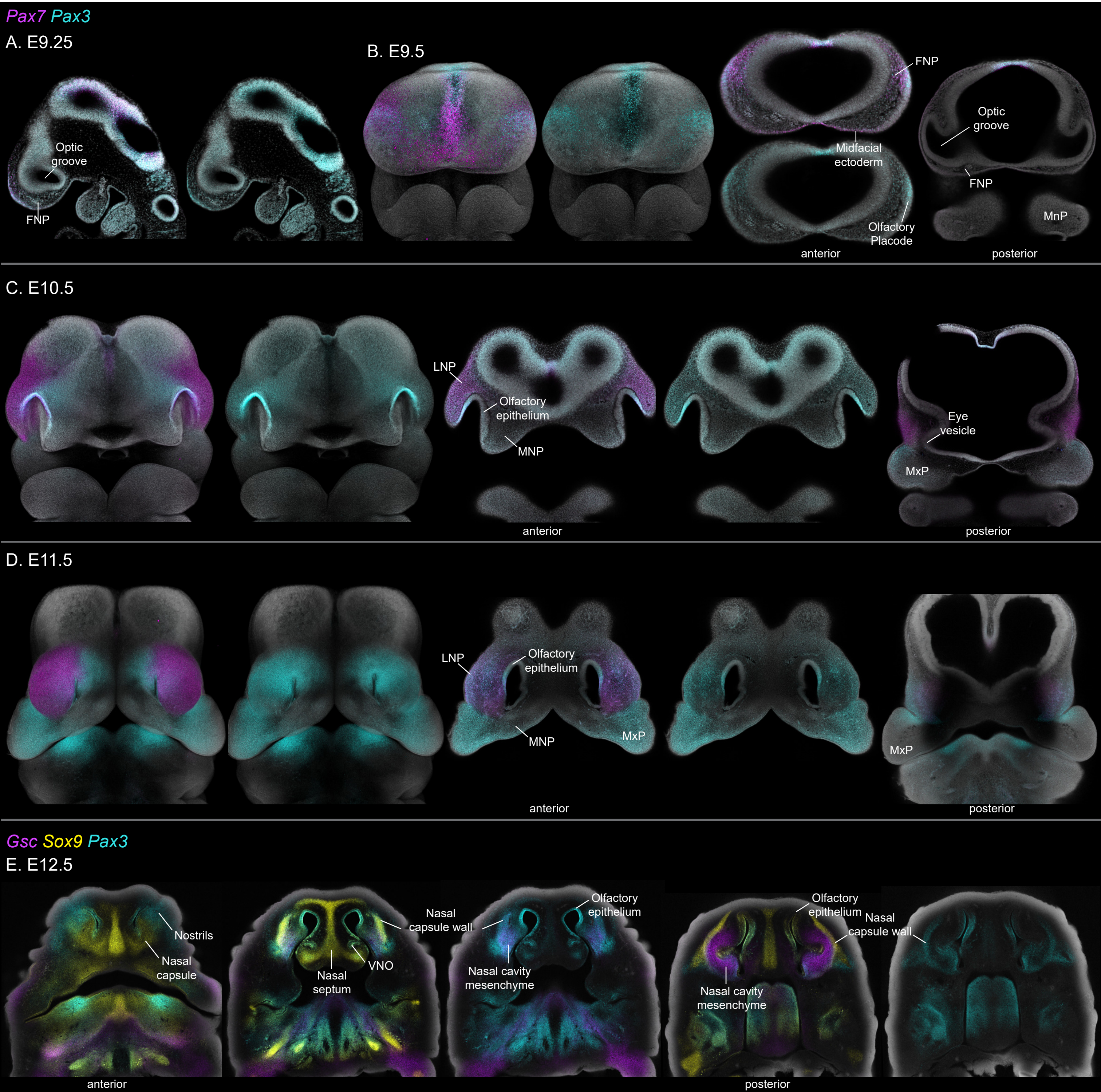

Supplementary Figure 23. ***Pax3*** **is mainly expressed in the ectoderm before E11.5.**

A-B) *Pax3* expression is observed in the face ectoderm and brain at (A) E9.25 and in the olfactory placode at (B) E9.5. Sparse *Pax3*-expressing cells in the dorsal-anterior frontonasal mesenchyme, colocalizing with Pax7. C) At E10.5, *Pax3* is observed in the LNP mesenchyme, although weaker compared to the expression observed in the developing olfactory epithelium. *Pax3* is also expressed in the dorsal-posterior MxP (dMxP). D) At E11.5, *Pax3* expression substantially increases in the MxP and the LNP mesenchyme. E) At E12.5, *Pax3* is expressed in the mesenchyme of the nostrils, in the walls of the cartilaginous nasal capsule, colocalizing here with *Sox9*, and in the antero-ventral nasal cavity mesenchyme colocalizing here with *Gsc*.

FNP, frontonasal prominence; LNP, lateral nasal prominence; MnP, mandibular prominence; MNP, medial nasal prominence; MxP, maxillary prominence; VNO, vomeronasal organ.

Supplementary Figure 24

MGI Abnormal facial prominence development in mouse

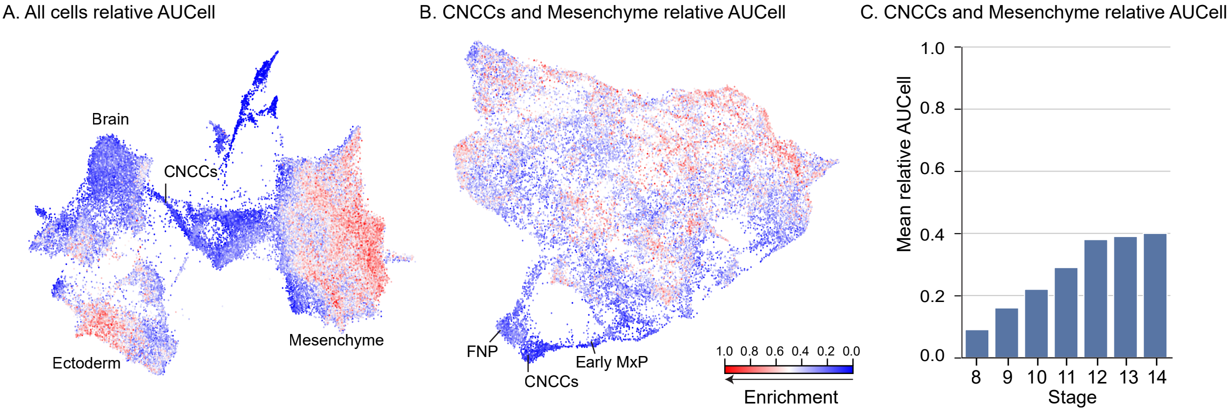

Supplementary Figure 24. Genes linked to abnormal facial prominence development in mouse are enriched in the brain, ectoderm and mesenchyme.

Relative AUCell scores for genes associated with abnormal facial prominence development in mouse, retrieved from the Mouse Genomic Informatics (MGI) using the search term "abnormal craniofacial development" (See Supp. Data 7 and 8), visualized on the UMAP for all cells (A), the cranial neural crest cells (CNCCs) and facial mesenchyme subset (B). Note the enrichment in the brain, ectoderm and mesenchyme clusters in (A). C) Mean relative AUCell score for genes associated with abnormal facial prominence development per stage in CNCCs and facial mesenchyme populations shown in (B).

Supplementary Figure 25

Enrichment patterns of genes associated with human craniofacial abnormalities (DISGENET)

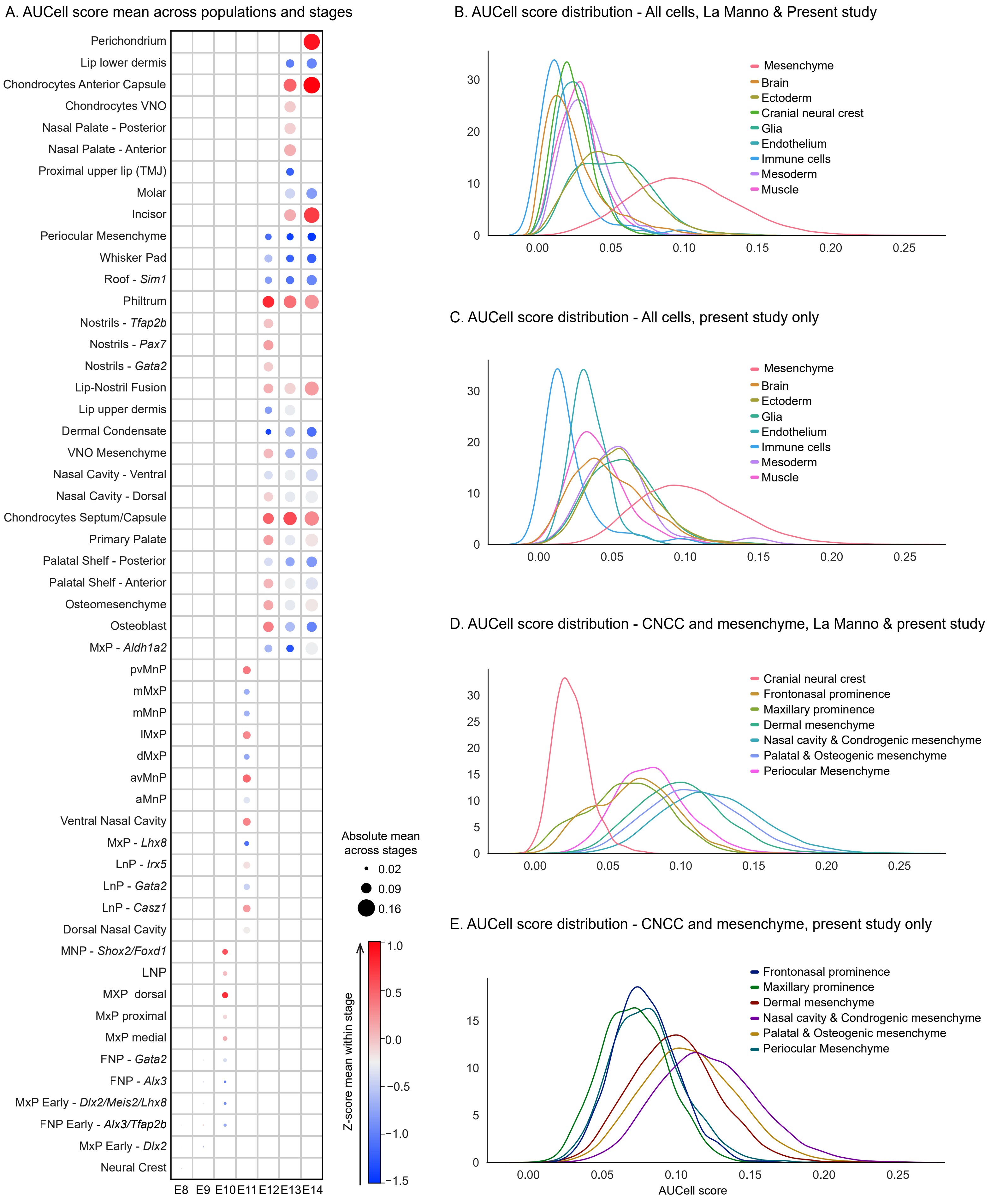

Supplementary Figure 25. Enrichment patterns of genes associated with abnormal craniofacial development in humans.

A) Dot plot of AUCell scores for the abnormal craniofacial development gene set (Supp. Data 6), across mesenchymal populations and developmental stages. Columns correspond to developmental stages and rows to cell populations (*i.e.* clusters). The size of the dot represents the mean AUCell score in that population at that developmental stage. The color of the dot indicates the stage-wise Z-score (standard deviation to the mean), highlighting enrichment differences among populations within a given stage (*e.g.* the redder the circle, the greater the enrichment of the gene set in that population compared to others in the same stage). It is important to note the size of the dot when comparing populations across stages. B) Density curve plot showing the AUCell score distribution between cell types, using both datasets (La Manno and present study). C) Similar to (B), using only the cells from the present study dataset, ruling out any batch effect in the density distribution pattern. D-E), similar to (B) and (C), respectively, showing the distribution of AUCell scores in the cranial neural crest cells (CNCCs) and mesenchyme subset.

Supplementary Table 1

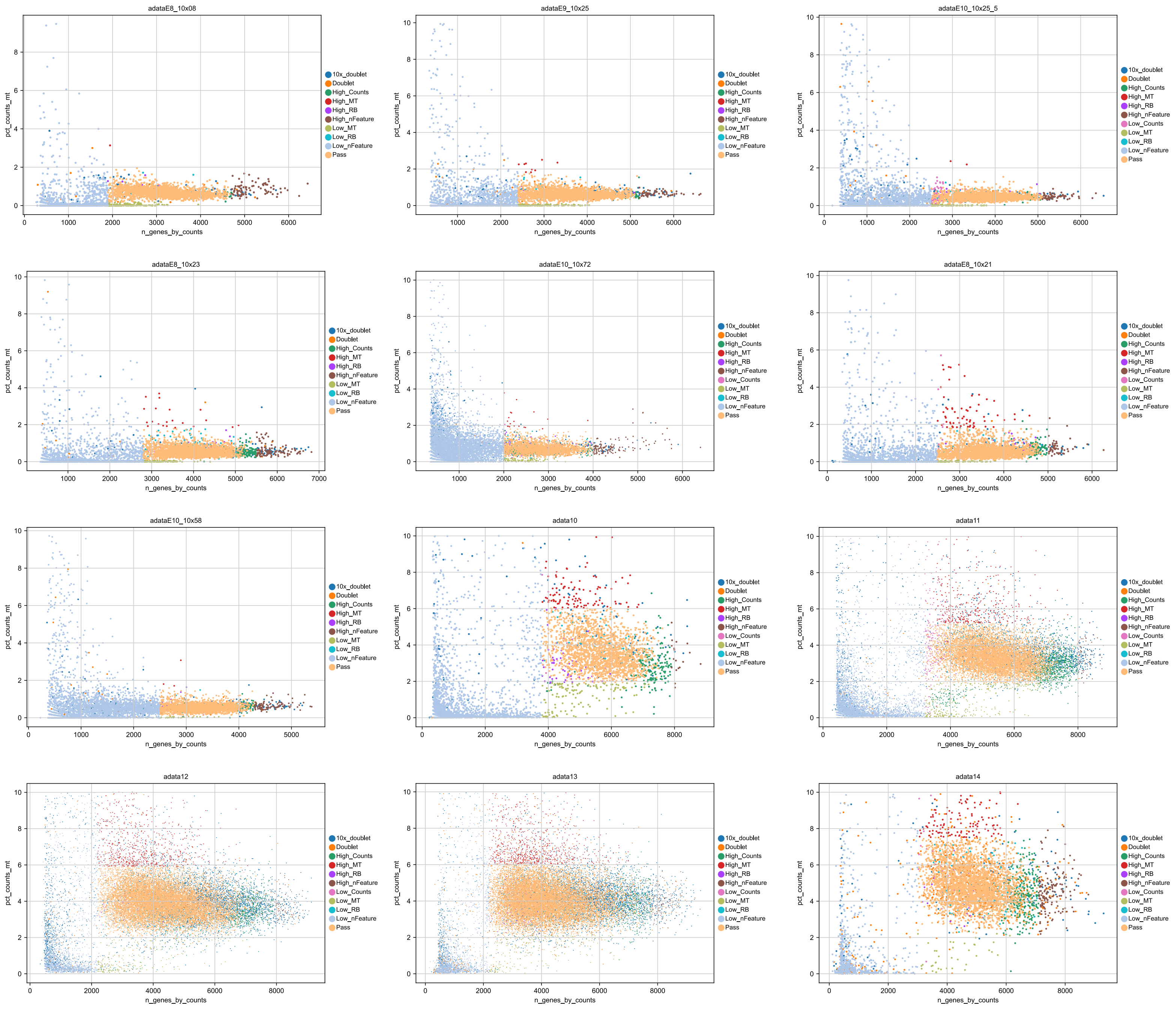

Supplementary Table 1. Overview of Quality Check (QC) strategies.

Scatter plots highlight the cells that passed QC by sample. The x-axis corresponds to the number of unique genes found per cell. The y-axis shows the percentage of mitochondrial counts per cell. Passing cells correspond to the tight light orange cluster in the middle of the plot. Non-passing cells are colored based on the reason why they failed the QC. QC was performed per sample, with cutoff values tailored to each sample. In many cases, non-passing cells failed in multiple categories. In those instances, only the first failed category is colored. Doublets were predicted using scrublet (https://github.com/swolock/scrublet).

Supplementary Table 2

Gene	HCR Amplifier	Target Sequence Accession Number	Comment
Cpne4	B1	NM_028719	
Cyp26b1	B1	NM_175475	
Dlx2	B1	NM_010054.3	
Dlx5	B1	NM_010056	
En1	B1	NM_010133	CCDS15235.1
Foxd1	B1	NM_008242	CCDS36759.1
Foxl2	B1	NM_012020	
Gsc	B1	NM_010351	
Mecom	B1	NM_001361034	
Pax7	B1	NM_011039.3	
Runx3	B1	ENSMUST00000056977.14	
Shh	B1	NM_009170	
Shox2	B1	NM_013665.1	
Sox18	B1	NM_009236	
Wnt6	B1	NM_009526.3	
Tbx15	B1	ENSMUST00000029462.10	
Tbx18	B2	NM_023814.4	
Casz1	B2	ENSMUST00000122222.8	
Fgf8	B2	NM_010205.3	
Gata2	B2	NM_008090	
Lef1	B2	NM_010703.5	
Lgr5	B2	NM_010195	
Meis2	B2	NM_001136072.2	CCDS50667.1
Meox2	B2	NM_008584	
Pou3f3	B2	NM_008900	
Sox9	B2	ENSMUST00000000579.3	
Sp5	B2	NM_022435	
Sp7	B2	NM_130458.4	
Tfap2b	B2	NM_009334.4	
Wnt3	B2	NM_009521.3	
Eya4	B3	NM_001347372.2	
Flrt2	B3	NM_201518.4	
Alcam	B4	NM_009655	CCDS37356.1
Aldh1a2	B4	NM_009022	
Alx3	B4	ENSMUST00000014747.3	
Bmp4	B4	NM_007554	
Car2	B4	ENSMUST00000029078.9	
Ccn2	B4	NM_010217	
Dlk1	B4	ENSMUST00000056110.15	
Ebf3	B4	NM_001113415.2	CCDS52422.1
Irx5	B4	NM_018826	
Lhx8	B4	NM_010713	CCDS17926.1
Lrriq1	B4	NM_001163559.2	
Nr2f2	B4	NM_009697.3	
Pax3	B4	NM_008781.4	
Pax9	B4	ENSMUST00000001538.10	
Pitx2	B4	NM_001042504.2	
Sim1	B4	NM_011376.3	CCDS48556.1
Six1	B4	NM_009189.3	
Sox21	B4	NM_177753.3	
Tbx22	B4	ENSMUST00000168174.9	
Wnt16	B4	ENSMUST00000031681.10	
Wnt5a	B4	NM_009524.4	

Supplementary Table 2. HCR probe information.

Target transcript sequences used to design the probe sets are provided. Amplifiers were coupled as follows: B1-647, B2-546, B3-488 and B4-594. Probe set stock concentration: 1µM
