# Supplementary material for: Positional programs in early murine facial development and their role in human facial shape variability
Source: Nat Commun. 2025 Nov 18;16:10112. doi: 10.1038/s41467-025-66017-y (PMC12627740; doi:10.1038/s41467-025-66017-y)
Supplement: Supplementary file 3 — Description of Additional Supplementary Files [file 41467_2025_66017_MOESM3_ESM.pdf]

File Name: Supplementary Data 1.

Description: CellChat results.

CellChat models the communication probability based on the law of mass action. Significant interactions are then identified on the basis of a statistical test, which randomly permutes the group labels of cells and then recalculates the communication probability. For each sample (in our particular case, developmental stage) CellChat returns the significant interactions that each group of cells (clusters) is modeled to have with other groups of cells, the probability of this interaction and the p-value. The probability values are relative within each sample and do not necessarily sum up to one. "Pathways" contains the probability of all significant communication at the interaction level (individual ligand/receptor pairs), and the associated p-value (values below 0.01 are returned as 0). "Interactions" contains the aggregated probability at the pathway level, where the probability of interactions belonging to a certain pathway is aggregated. "Pathway overview" contains an overview of each pathway and the ligand/receptor pairs associated with that pathway in the CellChat database. P-values were calculated using one-sided permutation test,using 100 permutations by default.

File Name: Supplementary Data 2.

Description: Relative contribution of each main signaling category (ECM, cell-cell contact and secreted signaling), including MK/PTN, to incoming and outgoing signaling per stage.

Values used to create Figure 4A, representing the relative incoming and outgoing signaling probability of each significant pathway per cell type (mesenchyme or ectoderm), per stage. The table includes values below 0.2%, which are merged (unlabeled bar sections) in Figure 4A.

File Name: Supplementary Data 3.

Description: Relative contribution of each main signaling category (ECM, cell-cell contact and secreted signaling) to incoming and outgoing signaling per stage.

Values used to create Figure 4B, representing the relative incoming and outgoing signaling probability of each significant pathway per cell type (mesenchyme or ectoderm), per stage, excluding the MK/PTN pathways. The table includes values below 0.2%, which are merged (unlabeled bar sections) in Figure 4B.

File Name: Supplementary Data 4.

Description: Genes associated with normal facial shape variation in humans.

List of genes associated with EFO0007841 (Facial morphology measurement) and their expression values in the cranial neural crest and mesenchyme populations, in the integrated dataset (all cells) and per stage (E8-E14). Tables present the genes, the associated GWAS variant (SNP) (RsID), the source study (studyID) from the GWAS Catalog (https://www.ebi.ac.uk/gwas/), the Open Targets score (score), the EFO term with the GWAS variants are associated with, the facial segment and corresponding human facial feature associated with the genetic variant found in the source study, the cluster with the highest log fold change in our data (Top Cluster), the Localized Marker score (LMD; lower scores indicate more localized expression) and the log fold change for each cluster. Log fold changes are only shown if relevant (based on rank_genes_groups score; see methods). Genes with an LMD score below the knee point (*i.e.* those showing spatially-restricted expression, see methods) are marked in green. Human facial features highlighted in yellow correspond to those matching an equivalent mouse cluster based on gene log fold change, also marked in yellow. Human features highlighted in orange are those not matching an equivalent mouse cluster, while those in red are human features with no equivalent mouse cluster in our dataset (*e.g.* mandible). Examples of spatially restricted genes in mouse (green) with matching human facial features (yellow) are shown in Fig. 7A-C.

File Name: Supplementary Data 5.

Description: List of genes used for AUCell analysis.

Genes associated with EFO term 0007841- Facial morphology measurement, were used to create Figure 6B-D. This gene set is the same presented in Supp. Table 4. Genes associated with Generalized Epilepsy (EFO_00005917), Immune system disease (EFO_0000540), Autosomal dominant intermediate Charcot-Marie Tooth disease (MONDO 0019548) and Keratosis (EFO_1000720), used to create Supplementary Figure 22C-F. Genes linked to abnormal craniofacial development in humans, retrieved from DISGENET (see methods). This gene set is the same presented in Supp. Data 6 and Figure 6E-G. Genes associated with the MGI term Abnormal facial prominence development were used to produce Supp. Fig. 24A- C.

Description: File Name: Supplementary Data 6.

Genes associated with abnormal craniofacial development in humans.

List of genes linked to abnormal craniofacial development in humans, retrieved from DISGENET (see methods), and their expression values in the cranial neural crest and mesenchyme populations, in the integrated dataset (all cells) and per stage (E8-14). The tables present the genes, the source study (PMID), the cluster with the highest log fold change in our data set (Top Cluster), the Localized Marker score (LMD; lower scores indicate more localized expression), and the log fold change for each cluster. Log fold changes are only shown if relevant (based on rank_genes_groups score; see methods). Genes with an LMD score below the knee point (*i.e.* those showing spatially-restricted expression, see methods) are marked in green.

File Name: Supplementary Data 7.

Description: Genes associated with abnormal facial prominence development in mouse.

List of genes linked to abnormal craniofacial development in humans, retrieved from the Mouse Genomic Informatics (MGI) database (see methods), and their expression values in the cranial neural crest and mesenchyme populations, in the integrated dataset (all cells) and per stage (E8-E14). The tables present the genes, gene accession number, the associated phenotype ID (https://monarchinitiative.org/), the cluster with the highest log fold change in present study dataset (Top Cluster), the Localized Marker score (LMD; lower scores indicate more localized expression), and the log fold change for each cluster. Log fold changes are only shown if relevant (based on rank_genes_groups score; see methods). Genes with an LMD score below the knee point (*e.g.* indicating more localized expression, see methods) are marked in green.

File Name: Supplementary Data 8.

Description: Disruption of early positional genes affects craniofacial development in mouse and humans.

Selected early positional genes expressed in mesenchymal populations of the present study single-cell transcriptomic data and their reported effects on craniofacial formation in mouse and human. The expression pattern of each gene is briefly described, and the known effects of mutations on each gene are summarized. Expression pattern of selected genes is shown in Fig. 7 E-G.
